# Practice Patterns and Survival Outcomes of Immunotherapy for Metastatic Colorectal Cancer

**DOI:** 10.1001/jamanetworkopen.2025.1186

**Published:** 2025-03-20

**Authors:** Shahla Bari, Marco Matejcic, Richard D. Kim, Hao Xie, Ibrahim H. Sahin, Benjamin D. Powers, Jamie K. Teer, Timothy A. Chan, Seth I. Felder, Stephanie L. Schmit

**Affiliations:** 1 Department of Medical Oncology, Duke Cancer Institute, Durham, North Carolina; 2 Department of Biostatistics and Bioinformatics, H. Lee Moffitt Cancer Center and Research Institute, Tampa, Florida; 3 Department of Gastrointestinal Oncology, H. Lee Moffitt Cancer Center and Research Institute, Tampa, Florida; 4 Department of Oncology, Mayo Clinic, Rochester, Minnesota; 5 University of Pittsburgh School of Medicine, Pennsylvania; 6 Department of Surgery, University of Maryland School of Medicine, Baltimore; 7 University of Maryland Marlene and Stewart Greenebaum Comprehensive Cancer Center, Baltimore; 8 Center for Immunotherapy and Precision Immuno-Oncology, Cleveland Clinic, Cleveland, Ohio; 9 Genomic Medicine Institute, Cleveland Clinic, Cleveland, Ohio; 10 Department of Molecular Medicine, Cleveland Clinic Lerner College of Medicine of Case Western Reserve University, Cleveland, Ohio

## Abstract

**Importance:**

Immune checkpoint inhibitors (ICIs) have been approved for treatment of microsatellite instable (MSI-H) metastatic colorectal cancer (mCRC), but factors associated with receipt and efficacy of ICIs in routine clinical practice remain largely unknown.

**Objective:**

To identify factors associated with receipt of ICIs and associated survival outcomes among patients with mCRC in routine clinical practice.

**Design, Setting, and Participants:**

This population-based cohort study used deidentified data from a nationwide electronic health record–derived database to include 18 932 patients diagnosed with mCRC between January 2013 and June 2019. Population-based patients were diagnosed with de novo mCRC and had at least 2 documented clinical visits on or after the date of diagnosis. The study analyses were performed between September 2020 and April 2021.

**Exposure:**

Patients received ICI therapy and/or chemotherapy as part of a systemic treatment for mCRC.

**Main Outcomes and Measures:**

The outcomes were receipt of ICI therapy, overall survival (OS), and time to treatment discontinuation (TTD).

**Results:**

In this cohort study of 18 932 patients diagnosed with mCRC (10 537 [55.7%] male; 546 [2.9%] Asian, 2005 [10.6%] Black or African American, 1674 [8.8%] Hispanic, 12 338 [65.2%] White, 4043 [21.4%] unknown race or ethnicity; median [IQR] age at metastatic diagnosis, 64.6 [55.0-73.3] years), patients with MSI-H tumors had a significantly higher probability of receiving ICIs than those with microsatellite stable (MSS) tumors (odds ratio [OR], 22.66 [95% CI, 17.30-29.73]; *P* < .001), whereas patients initially diagnosed with synchronous mCRC had significantly lower odds of receiving ICIs than patients with metachronous mCRC (OR, 0.57 [95% CI, 0.45-0.73]; *P* < .001). Patients with MSI-H tumors who received ICIs as first line of therapy had significantly longer OS than those receiving chemotherapy only (HR, 0.37 [95% CI, 0.25-0.56]; *P* < .001). Among patients with MSS tumors, ICI-based therapy was associated with significantly longer OS for patients with high albumin level (vs low: HR, 0.28 [95% CI, 0.18-0.45]; *P* < .001) and antibiotic use (vs nonuse: HR, 0.43 [95% CI, 0.27-0.67]; *P* < .001), but a significantly shorter OS for patients with synchronous mCRC (vs metachronous: HR, 1.90 [95% CI, 1.24-2.89]; *P* = .003). In addition, 29 out of 235 patients with MSS tumors (12.3%) experienced durable responses on ICI-based therapy. Similar patterns of associations with TTD were observed.

**Conclusions and Relevance:**

In this cohort study of patients with mCRC, clinical characteristics were associated with different survival outcomes in patients treated with ICI-based therapy, with important clinical implications for patients with MSS tumors who are generally unresponsive to immunotherapy.

## Key Points

**Question:**

What factors are associated with receipt of immune checkpoint inhibitors (ICIs) and survival outcomes in patients with metastatic colorectal cancer (mCRC) in routine clinical practice?

**Findings:**

This cohort study of 18 932 patients found a 63% higher probability of survival among patients with microsatellite instable (MSI-H) mCRC who received early ICI-based treatment compared with chemotherapy. ICIs were associated with 57% and 72% higher survival probability in patients with microsatellite stable (MSS) tumors using antibiotics and with high albumin levels, respectively.

**Meaning:**

This study supports findings from clinical trials and identifies factors that may influence clinical outcomes associated with ICIs in patients with MSS mCRC.

## Teaser

This cohort study evaluates factors associated with receipt of immune checkpoint inhibitors and survival outcomes among patients with metastatic colorectal cancer (CRC) treated in routine clinical practice.

## Introduction

Approximately 25% of patients with colorectal cancer (CRC) have metastatic disease at initial diagnosis, and an additional 25% who present with clinically local-regional disease develop metastases.^1^ In recent years, clinical trials have shown that treatment with immune checkpoint inhibitors (ICIs) provides durable response as well as longer survival in patients with microsatellite instable (MSI-H) mCRC that are refractory to conventional treatment.^2,3,4,5^ These results led to the accelerated approval of ICIs for refractory MSI-H mCRC by the US Food and Drug Administration (FDA) in 2017,^6^ followed by approval as first-line treatment in 2020.^7^ However, clinical trials are often limited by modest sample size and strict inclusion criteria; larger studies using data from routine clinical care are needed to confirm the effectiveness of ICIs in a routine clinical practice setting.^8,9^ In addition, overall survival (OS) has limitations in assessing the safety and efficacy of ICIs because it inherently captures the impact of all therapies administered to the patient before or after the ICI-containing regimen as well as competing causes of death. Time to treatment discontinuation (TTD), which reflects the period patients may have derived benefit from the intervention, has been increasingly used in routine clinical practice studies as a surrogate of OS and may serve as a better proxy of therapeutic index.^10,11^

Using annotated and/or audited electronic health record (EHR) data from the Flatiron Health database, we conducted a retrospective study of 23 963 patients with mCRC to identify factors associated with receipt of ICIs compared with conventional treatment in US oncology practices. In addition, we investigated factors that may be associated with OS and TTD in patients treated with ICIs.

## Methods

### Data Source and Patient Selection

A total of 23 963 patients with mCRC were selected from the nationwide Flatiron Health electronic health record (EHR)-derived deidentified database.^12^ Patients were diagnosed with mCRC and had at least 2 documented clinical visits on or after January 1, 2013, through June 31, 2019, with follow-up through December 31, 2019. A total of 18 932 patients from the routine clinical practice cohort who received treatment for mCRC met eligibility criteria and were selected for this study. Details on the Flatiron Health database and patient selection are presented in the eMethods of Supplement 1, and a flowchart of sample selection and sample size used in each analysis is presented in the eFigure in Supplement 1. The study protocol was deemed not to be human participants research by the Moffitt Cancer Center scientific review committee. Informed consent was not required as this was a noninterventional study using deidentified patient records. The reporting of results in this cohort study adhere to the Strengthening the Reporting of Observational Studies in Epidemiology (STROBE) reporting guideline.

### Data Collection

Baseline demographic, clinical, and tumor characteristics of patients included birth year, primary cancer site, disease stage at initial diagnosis, date of metastatic diagnosis, sex, race, ethnicity, tumor mutational status for *KRAS*, *BRAF*, and *NRAS* genes, mismatch repair (MMR) status, insurance status at initial diagnosis, practice type (academic or community), Eastern Cooperative Oncology Group (ECOG) performance status, blood albumin level, and antibiotic and proton pump inhibitor (PPI) use. Patient race (Asian, Black or African American, White) and ethnicity (Hispanic/Latino, non-Hispanic/non-Latino) data were collected from patients via EHR, survey, and self-report. This information was recorded directly into the EHR or in the practice management system, which was later imported into the EHR. ECOG performance status and blood albumin level were recorded within 3 months prior to or after immunotherapy initiation. Antibiotic and proton pump inhibitor (PPI) use was recorded from 1 month before until the end of immunotherapy. Individual antibiotic classes and breakdown of patients receiving these drugs in each class are presented in eTable 1 in Supplement 1. Details on measurement and categorization of each variable are presented in Supplement 1.

Date of death was identified using structured and unstructured EHR-derived data such as clinician notes and condolence letters, external death data sources from the US Social Security Death Index,^13^ and a commercial death dataset that mines data from obituaries, funeral homes, and other sources.^14^ Death dates were available at month- and year-level granularity, and the 15th of each month was used for analytical purposes.

### Clinical End Points

ICI-based therapy was defined as the administration of any of the following drugs during treatment of mCRC: nivolumab, pembrolizumab, atezolizumab, ipilimumab, tremelimumab, durvalumab, or avelumab. Primary outcomes were receipt of ICI-based therapy (yes/no) and OS, defined as the time from index date to date of death, last known follow-up, or end of study period (December 31, 2019), whichever occurred first. The secondary outcome was TTD, defined as the time from the first to the last episode of treatment regimen, last known follow-up, or death, whichever occurred first. Index date (date on which patients received their first treatment administration) was defined differently based on group comparison in survival models (eMethods in Supplement 1). Censoring was carried out using medication, mortality, and visit information (eMethods in Supplement 1).

### Statistical Analysis

The primary study analyses were conducted between September 2020 and April 2021. Descriptive statistics were used to summarize baseline patient characteristics by receipt of ICI-based therapy (no vs yes) and by MMR status (MSS vs MSI-H). Between-group differences were assessed using the Kruskal-Wallis test for numerical variables, and χ^2^ test for categorical variables.

Multivariable logistic regression was used to estimate odds ratios (OR) and 95% CIs for factors associated with receipt of ICIs. We also analyzed receipt of ICI-based therapy stratified by MMR status. The following covariates were preselected based on clinical knowledge and included in multivariable models because of statistically significant associations in univariate tests of independence (Kruskal-Wallis test or χ^2^ test *P* < .05): sex (female vs male), stage at initial diagnosis (I-III vs IV), primary cancer site (colon vs rectum), MMR status (MSS vs MSI-H), and *BRAF* mutation status (wild-type vs positive).

The Kaplan-Meier method and log-rank test were used to estimate differences in median time to event (death for OS; treatment discontinuation for TTD) between prespecified subgroups. The correlation between OS and TTD among patients treated with ICIs was assessed through the Spearman rank correlation coefficient (ρ) and 95% CIs. The correlation analysis was restricted to patients who died during the study period.

Multivariable Cox proportional hazards regression was used to estimate hazard ratios (HRs) and 95% CIs for the associations between patient characteristics and the event of interest (OS or TTD). We also analyzed clinical outcomes stratified by MMR status. The following covariates were preselected based on clinical knowledge and included in the multivariable models because of statistically significant associations in univariable models (log-rank test *P* < .05): sex (female vs male), stage at initial diagnosis (I-III vs IV), primary cancer site (colon vs rectum), MMR status (MSS vs MSI-H), *KRAS* mutation status (wild-type vs positive), immunotherapy as first-line treatment (no vs yes), immunotherapy plus chemotherapy (no vs yes), ECOG performance status (0-1 vs 2-4), albumin levels (<3 g/dL vs ≥3 g/dL [SI conversion factor: to convert albumin to g/dL, multiply by 10]), and antibiotic use (no vs yes).

Patients with missing values for any of the covariates were excluded from multivariable models. However, sensitivity analyses were conducted to evaluate the robustness of the analytical approach and confirm the results obtained in the main sample (see details in Supplement 1).

Statistical significance was assessed at 2-sided *P* < .05. All analyses were performed using R version 3.5.0 (R Project for Statistical Computing) from September 2020 to April 2021. Flatiron Health Inc did not participate in the analysis of the data.

## Results

Among a total of 18 932 patients included in this study, 10 537 (55.7%) were male; 546 (2.9%) were Asian, 2005 (10.6%) were Black or African American, 1674 (8.8%) were Hispanic, 12 338 (65.2%) were White, 4043 (21.4%) had unknown race or ethnicity; and the median (IQR) age at metastatic diagnosis was 64.6 (55.0-73.3) years (eTable 2 in Supplement 1). The median (IQR) follow-up period from the date of mCRC diagnosis to either date of death, last observed follow-up, or end of study period was 16.7 (8.3-29.5) months.

### Factors Associated With Receipt of Immunotherapy

Of the 18 932 patients selected for this study, 566 patients (3.0%) received ICIs at some point during treatment. Among the 6451 patients (34.1%) diagnosed with mCRC after FDA approval of ICIs for treatment of refractory MSI-H mCRC, 220 patients (3.4%) received ICIs at some point during treatment (eTable 3 in Supplement 1).

Patient characteristics stratified by receipt of ICI-based therapy are summarized in eTable 2 in Supplement 1. A significantly higher likelihood of receiving ICIs was observed among female patients (vs males), patients initially diagnosed at stage I-III (vs stage IV), with colon cancer (vs rectal cancer), MSI-H tumors (vs MSS tumors), *BRAF* mutations (vs wild-type), or diagnosed after FDA approval (vs before). No significant difference in immunotherapy receipt was found by race, ethnicity, insurance status at diagnosis, practice type, age at metastatic diagnosis, and *KRAS* or *NRAS* mutation status.

In a multivariable logistic regression model (Table 1), patients with MSI-H tumors had 22-fold higher odds of receiving ICIs than those with MSS tumors (OR, 22.66 [95% CI, 17.30-29.73]; *P* < .001). Patients diagnosed with synchronous mCRC (ie, stage IV at initial diagnosis) had significantly lower odds of receiving ICIs compared with patients with metachronous mCRC (OR, 0.57 [95% CI, 0.45-0.73]; *P* < .001), and the difference remained statistically significant in both the MSI-H cohort (OR, 0.49 [95% CI, 0.34-0.72]; *P* < .001) and the MSS cohort (OR, 0.64 [95% CI, 0.46-0.89]; *P* = .007). Estimates for the association between patient characteristics and immunotherapy receipt restricted to patients diagnosed with mCRC after FDA approval (n = 6421) were similar with those in the overall patient population in terms of magnitude, directionality, and statistical significance (eTable 4 in Supplement 1).

**Table 1.** Multivariable Logistic Regression Models for Association Between Characteristics of Patients With Metastatic Colorectal Cancer and Receipt of Immunotherapy

Characteristics	All patients (n = 7412)^a^^,^^b^^,^^c^				MSI-H tumor carriers (n = 508)				MSS tumor carriers (n = 6904)			
	Immunotherapy, No.		Adjusted OR (95% CI)^d^	*P* value	Immunotherapy, No.		Adjusted OR (95% CI)	*P* value	Immunotherapy, No.		Adjusted OR (95% CI)	*P* value
	No	Yes			No	Yes			No	Yes		
Sex												
Female	3123	170	1 [Reference]	.19	163	99	1 [Reference]	.25	2060	71	1 [Reference]	.45
Male	3957	162	0.84 (0.66-1.09)		164	82	0.80 (0.54-1.18)		3793	80	0.88 (0.64-1.22)	
Disease stage at initial diagnosis^e^												
I-III	2629	182	1 [Reference]	<.001	144	111	1 [Reference]	<.001	2485	71	1 [Reference]	.007
IV	4451	150	0.57 (0.45-0.73)		183	70	0.49 (0.34-0.72)		4268	80	0.64 (0.46-0.89)	
Primary cancer site												
Colon	5456	288	1 [Reference]	.37	307	168	1 [Reference]	.70	5149	120	1 [Reference]	.26
Rectum	1624	44	0.85 (0.60-1.21)		20	13	1.16 (0.55-2.45)		1604	31	0.79 (0.53-1.19)	
MMR status												
MSS	6753	151	1 [Reference]	<.001	NA	NA	1 [Reference]	-NA	NA	NA	1 [Reference]	NA
MSI-H	327	181	22.66 (17.30-29.73)		NA	NA	NA		NA	NA	NA	
*BRAF* mutation status												
Wild-type	6364	240	1 [Reference]	.97	191	103	1 [Reference]	.98	6173	137	1 [Reference]	.89
Positive	716	92	1.01 (0.73-1.38)		136	78	0.99 (0.67-1.48)		580	14	1.04 (0.60-1.82)	

Abbreviations: MMR, mismatch repair; MSI-H, microsatellite instable; MSS, microsatellite stable; NA, not applicable; OR, odds ratio.

^a^ Number of patients retained in the multivariate models.

^b^ Sample includes patients diagnosed with metastatic colorectal cancer between January 1, 2015, and December 31, 2019.

^c^ Sample includes patients with unknown MMR status.

^d^ Models were adjusted for sex, stage at initial diagnosis, primary cancer site, MMR status, and BRAF mutation status.

^e^ All patients in the cohort were either diagnosed with metastatic colorectal cancer or had progressed to metastatic disease after early-stage diagnosis.

### Patterns of Immunotherapy Treatment

Treatment patterns and performance status of patients who received ICI-based therapy (n = 566) are presented in eTable 5 in Supplement 1. Compared to patients with MSS tumors (n = 235), those with MSI-H tumors (n = 234) had a significantly higher likelihood of receiving ICIs as first-line treatment (80 [34.2%] vs 28 [11.9%] for MSS cohort; *P* < .001) or as monotherapy (219 [93.6%] vs 174 [74.0%] for MSS cohort; *P* < .001). In addition, patients with MSI-H tumors had a significantly higher median age at metastatic diagnosis (66.5 vs 60.4 years for MSS cohort, *P* < .001) and were significantly more likely to have metachronous mCRC (135 [57.7%] vs 45 [46.4%] for MSS cohort; *P* = .02). Patterns of immunotherapy treatment were similar among patients diagnosed with mCRC after FDA approval, except for a lack of statistically significant difference in median age at metastatic diagnosis by MMR status (eTable 6 in Supplement 1).

### Association of Immunotherapy With Clinical Outcomes

**Figure 1.**
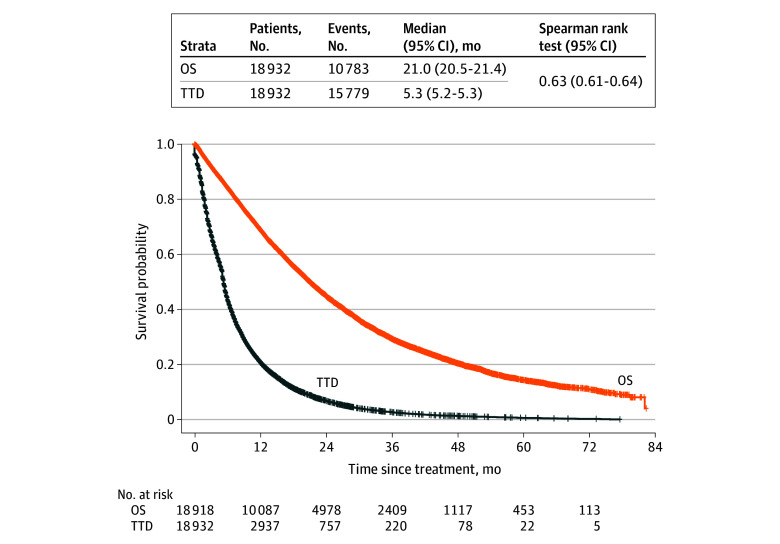
Kaplan-Meier Survival Curves for Overall Survival (OS) and Time to Treatment Discontinuation (TTD) in All Patients With Metastatic Colorectal Cancer Index date was the first episode of any systemic treatment regimen administered on or after the date of metastatic disease being diagnosed. For patients who died during the study period (n = 10 783), the correlation between OS and TTD was estimated using the Spearman rank test: ρ = 0.63 (95% CI, 0.61-0.64).

Figure 1 shows Kaplan-Meier survival curves for OS and TTD in all patients with mCRC (n = 18 932). The median OS was 21.0 (95% CI, 20.5-21.4) months, while the median TTD was 5.3 (95% CI, 5.2-5.3) months. For patients who died during the study period (n = 10 783), the correlation between OS and TTD was ρ = 0.63 (95% CI, 0.61-0.64).

The associations between receipt of immunotherapy (vs chemotherapy only) and clinical outcomes were statistically significantly different between the MSI-H and MSS cohorts (OS *P* for interaction = .002; TTD *P* for interaction = .03). Survival estimates by line of immunotherapy receipt are shown in eTable 7 in Supplement 1. Compared with patients treated with chemotherapy only (no immunotherapy), a significantly longer OS was observed for patients who received ICIs as first line of therapy (HR, 0.60 [95% CI, 0.44-0.82]; *P* = .002), but not for those who received ICIs as second or later line of therapy. When patients were stratified by MMR status, those with MSI-H tumors had a significantly longer OS associated with first line of ICI-based therapy compared with chemotherapy (HR, 0.37 [95% CI, 0.25-0.56]; *P* < .001) whereas no association was found in the MSS cohort. Line of immunotherapy receipt was not associated with TTD in the MSI-H cohort.

### Factors Associated With Response to Immunotherapy

**Figure 2.**
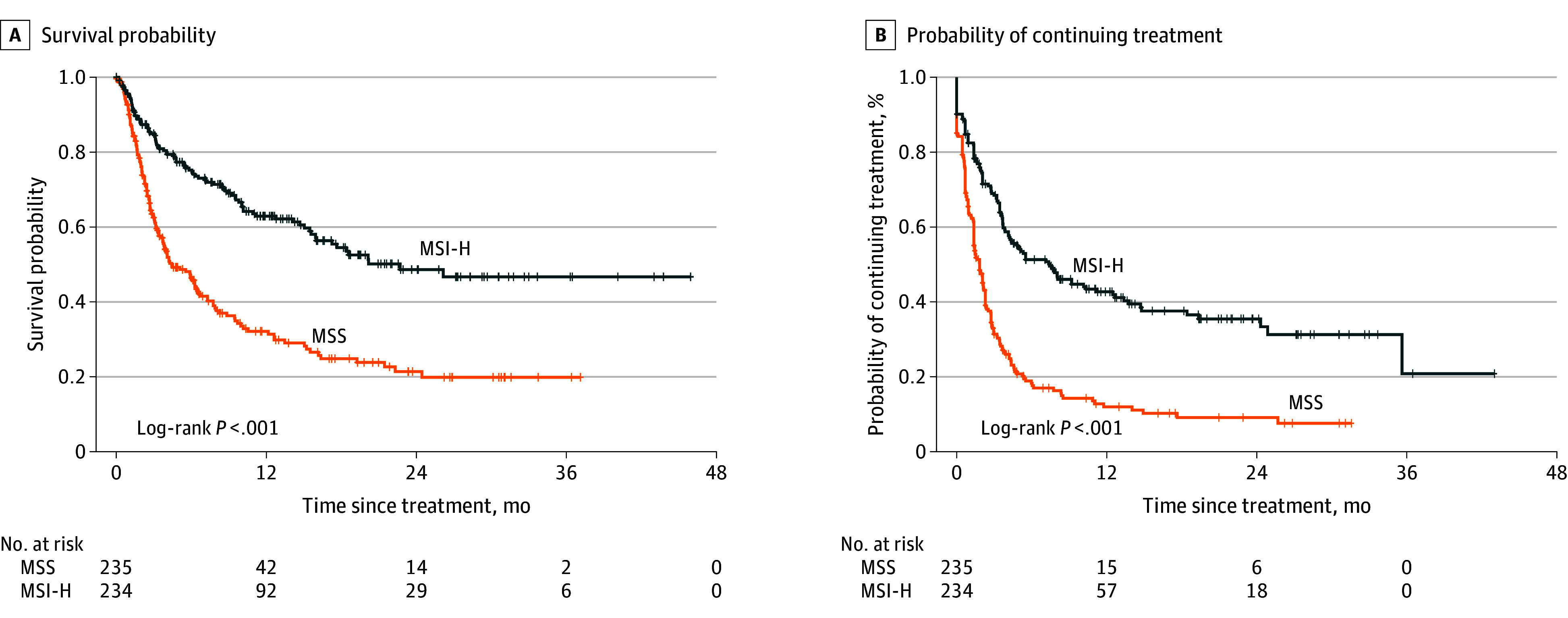
Kaplan-Meier Curves for Overall Survival (OS) and Time to Treatment Discontinuation (TTD) by Microsatellite Stability Status Among Patients With Metastatic Colorectal Cancer Treated With Immunotherapy Index date was the start of immune checkpoint inhibitor (ICI)-based therapy defined as the first administration or noncancelled order of any of the following drugs: nivolumab, pembrolizumab, atezolizumab, ipilimumab, tremelimumab, durvalumab, or avelumab. A, Patients with microsatellite instable (MSI-H) tumors had a significantly reduced hazard of death (overall survival log-rank *P* < .001) compared with patients with microsatellite stable (MSS) tumors. B, Patients with MSI-H tumors had a significantly lower likelihood of immunotherapy discontinuation (TTD log-rank *P* < .001) relative to patients with MSS tumors.

Median OS and TTD estimates stratified by characteristics of patients treated with ICIs (n = 566) are shown in eTable 8 in Supplement 1, and Kaplan-Meier curves by MMR status are presented in Figure 2. Among immunotherapy-treated patients, those with MSI-H tumors had a significantly reduced hazard of death (OS log-rank *P* < .001) and a lower likelihood of immunotherapy discontinuation (TTD log-rank *P* < .001) relative to patients with MSS tumors.

Results from multivariable Cox proportional hazards models for OS and TTD among patients who received ICI-based therapy are shown in Table 2 and Table 3, respectively. For OS, patients with synchronous mCRC had a significantly higher hazard of death than patients with metachronous mCRC (HR, 1.65 [95% CI, 1.19-2.29]; *P* = .003), while the hazard of death was significantly lower in patients with MSI-H tumors (vs MSS: HR, 0.32 [95% CI, 0.22-0.47]; *P* < .001), high albumin level (vs low level: HR, 0.37 [95% CI, 0.25-0.54]; *P* < .001) and antibiotic use (vs nonuse: HR, 0.51 [95% CI, 0.36-0.74]; *P* < .001). In the MSI-H cohort, a significantly higher hazard of death was observed for males (vs females: HR, 2.97 [95% CI, 1.60-5.52]; *P* < .001) and patients with ECOG 2 to 4 (vs 0 to 1: HR, 2.3 [95% CI, 1.08-4.93]; *P* = .03). In the MSS cohort, the hazard of death was significantly higher in patients with synchronous mCRC (vs metachronous: HR, 1.90 [95% CI, 1.24-2.89]; *P* = .003), but significantly lower in patients with high albumin level (vs low level: HR, 0.28 [95% CI, 0.18-0.45]; *P* < .001) and who used antibiotics (vs nonuse: HR, 0.43 [95% CI, 0.27-0.67]; *P* < .001).

**Table 2.** Multivariable Cox Proportional Hazards Models for Differences in OS Among Patients With Metastatic Colorectal Cancer Treated With Immunotherapy^a^

Characteristics	All patients (161 events/307 total)^b^^,^^c^^,^^d^				MSI-H tumor carriers (52 events/146 total)				MSS tumor carriers (109 events/161 total)			
	Patients, No.	Events, No. (% total)^e^	Adjusted HR (95% CI)^f^	*P* value	Patients, No.	Events, No. (% total)	Adjusted HR (95% CI)	*P* value	Patients, No.	Events, No. (% total)	Adjusted HR (95% CI)	*P* value
**OS**												
Sex												
Female	147	67 (41.6)	1 [Reference]	.14	77	19 (36.5)	1 [Reference]	<.001	70	48 (44.0)	1 [Reference]	.92
Male	160	94 (58.4)	1.28 (0.92-1.76)		69	33 (63.5)	2.97 (1.6-5.52)		91	61 (56.0)	0.98 (0.66-1.46)	
Disease stage at initial diagnosis^g^												
I-III	168	77 (47.8)	1 [Reference]	.003	87	30 (57.7)	1 [Reference]	.28	81	47 (43.1)	1 [Reference]	.003
IV	139	84 (52.2)	1.65 (1.19-2.29)		59	22 (42.3)	1.39 (0.77-2.5)		80	62 (56.9)	1.90 (1.24-2.89)	
Primary cancer site												
Colon	257	128 (79.5)	1 [Reference]	.39	137	46 (88.5)	1 [Reference]	.10	120	82 (75.2)	1 [Reference]	.55
Rectum	50	33 (20.5)	1.2 (0.79-1.8)		9	6 (11.5)	2.15 (0.86-5.37)		41	27 (24.8)	1.16 (0.72-1.86)	
MMR status												
MSS	161	109 (67.7)	1 [Reference]	<.001	NA	NA	1 [Reference]	NA	NA	NA	1 [Reference]	NA
MSI-H	146	52 (32.3)	0.32 (0.22-0.47)		NA	NA	NA		NA	NA	NA	
*KRAS* status												
Wild-type	176	82 (50.9)	1 [Reference]	.99	105	38 (73.1)	1 [Reference]	.74	71	44 (40.4)	1 [Reference]	.88
Positive	131	79 (49.1)	1 (0.72-1.4)		41	14 (26.9)	0.9 (0.47-1.72)		90	65 (59.6)	0.97 (0.65-1.45)	
Immunotherapy as first treatment												
No	251	145 (90.1)	1 [Reference]	.11	103	40 (76.9)	1 [Reference]	.13	148	105 (96.3)	1 [Reference]	.30
Yes	56	16 (9.9)	0.63 (0.36-1.11)		43	12 (23.1)	0.58 (0.29-1.17)		13	4 (3.7)	0.57 (0.2-1.64)	
ECOG status^h^												
0-1	240	121 (75.2)	1 [Reference]	.19	111	35 (67.3)	1 [Reference]	.03	129	86 (78.9)	1 [Reference]	.52
2-4	67	40 (24.8)	1.29 (0.88-1.89)		35	17 (32.7)	2.3 (1.08-4.93)		32	23 (21.1)	1.17 (0.73-1.88)	
Albumin level^h^												
<3g/dL	52	38 (23.6)	1 [Reference]	<.001	24	12 (23.1)	1 [Reference]	.77	28	26 (23.9)	1 [Reference]	<.001
≥3g/dL	255	123 (76.4)	0.37 (0.25-0.54)		122	40 (76.9)	0.89 (0.39-2.03)		133	83 (76.1)	0.28 (0.18-0.45)	
Antibiotic use^i^												
No	209	117 (72.7)	1 [Reference]	<.001	101	38 (73.1)	1 [Reference]	.30	108	79 (72.5)	1 [Reference]	<.001
Yes	98	44 (27.3)	0.51 (0.36-0.74)		45	14 (26.9)	0.7 (0.35-1.37)		53	30 (27.5)	0.43 (0.27-0.67)	

Abbreviations: ECOG, Eastern Cooperative Oncology Group; HR, hazard ratio; ICI, immune checkpoint inhibitor; MMR, mismatch repair; MSI-H, microsatellite instable; MSS, microsatellite stable; OS, overall survival; PPI, proton pump inhibitor.

SI unit conversion: To convert albumin to g/L, multiply by 10.

^a^ Each patient had a diagnosis of metastatic colorectal cancer (*International Classification of Diseases, Ninth Revision [ICD-9]*: 153.x, 154.x; *ICD-10*: C18x, C19x, C20x, C21x) and at least 2 documented clinical visits between January 2013 and December 2019. Sensitivity analyses were carried out on patients diagnosed after Food and Drug Administration approval of ICIs for treatment of MSI-H metastatic colorectal cancer (May 2017).

^b^ Number of patients retained in the multivariate models.

^c^ Sample includes metastatic colorectal cancer patients who received immune checkpoint inhibitors between January 1, 2015, and December 31, 2019.

^d^ Sample includes patients with unknown MMR status.

^e^ Index date was the start of ICI-based therapy defined as the first administration or noncancelled order of any of the following drugs: nivolumab, pembrolizumab, atezolizumab, ipilimumab, tremelimumab, durvalumab, or avelumab.

^f^ For OS, models were adjusted for sex, stage at initial diagnosis, primary cancer site, MMR status, *KRAS* mutation status, immunotherapy as first-line treatment, ECOG performance status, albumin levels, and antibiotic use.

^g^ All patients in the cohort were either diagnosed with metastatic colorectal cancer or had progressed to metastatic disease after early-stage diagnosis.

^h^ ECOG status and albumin levels were recorded 3 months before or after immunotherapy initiation.

^i^ Antibiotics or PPIs taken anytime from 1 month before the start of immunotherapy until the end.

**Table 3.** Multivariable Cox Proportional Hazards Models for Differences in TTD Among Patients With Metastatic Colorectal Cancer Treated With Immunotherapy^a^

Characteristics	All patients (253 events/371 total)^b^^,^^c^^,^^d^				MSI-H tumor carriers (96 events/177 total)				MSS tumor carriers (157 events/194 total)			
	Patients, No.	Events, No. (% total)^e^	Adjusted HR (95% CI)^f^	*P* value	Patients, No.	Events, No. (% total)	Adjusted HR (95% CI)	*P* value	Patients, No.	Events, No. (% total)	Adjusted HR (95% CI)	*P* value
TTD												
Sex												
Female	185	121 (47.8)	1 [Reference]	.02	93	45 (46.9)	1 [Reference]	.02	92	76 (48.4)	1 [Reference]	.41
Male	186	132 (52.2)	1.34 (1.04-1.73)		84	51 (53.1)	1.67 (1.08-2.57)		102	81 (51.6)	1.15 (0.82-1.62)	
Disease stage at initial diagnosis												
I-III	196	120 (47.4)	1 [Reference]	<.001	102	53 (55.2)	1 [Reference]	.17	94	67 (42.7)	1 [Reference]	<.001
IV	175	133 (52.6)	1.74 (1.34-2.26)		75	43 (44.8)	1.37 (0.87-2.13)		100	90 (57.3)	1.92 (1.36-2.70)	
Primary cancer site												
Colon	313	208 (82.2)	1 [Reference]	.69	165	86 (89.6)	1 [Reference]	.06	148	122 (77.7)	1 [Reference]	.60
Rectum	58	45 (17.8)	1.07 (0.76-1.51)		12	10 (10.4)	1.93 (0.97-3.84)		46	35 (22.3)	0.90 (0.60-1.34)	
MMR status												
MSS	194	157 (62.1)	1 [Reference]	<.001	NA	NA	1 [Reference]	NA	NA	NA	1 [Reference]	NA
MSI-H	177	96 (37.9)	0.41 (0.30-0.55)		NA	NA	NA		NA	NA	NA	
*KRAS* status												
Wild-type	209	129 (51.0)	1 [Reference]	.40	126	65 (67.7)	1 [Reference]	.70	83	64 (40.8)	1 [Reference]	.88
Positive	162	124 (49.0)	1.12 (0.86-1.46)		51	31 (32.3)	1.09 (0.69-1.72)		111	93 (59.2)	1.03 (0.72-1.46)	
Immunotherapy as first treatment												
No	305	219 (86.6)	1 [Reference]	.57	128	71 74.0)	1 [Reference]	.57	177	148 (94.3)	1 [Reference]	.33
Yes	66	34 (13.4)	0.89 (0.60-1.32)		49	25 (26.0)	0.86 (0.52-1.44)		17	9 (5.7)	0.71 (0.35-1.46)	
Immunotherapy plus chemotherapy^g^												
No	314	202 (79.8)	1 [Reference]	.51	166	85 (88.5)	1 [Reference]	.047	148	117 (74.5)	1 [Reference]	.73
Yes	57	51 (20.2)	1.11 (0.81-1.54)		11	11 (11.5)	2.02 (1.01-4.03)		46	40 (25.5)	0.94 (0.65-1.36)	
Albumin level^h^												
<3g/dL	59	45 (17.8)	1 [Reference]	<.001	28	16 (16.7)	1 [Reference]	.38	31	29 (18.5)	1 [Reference]	<.001
≥3g/dL	312	208 (82.2)	0.54 (0.39-0.76)		149	80 (83.3)	0.78 (0.44-1.36)		163	128 (81.5)	0.44 (0.29-0.67)	
Antibiotic use^i^												
No	250	170 (67.2)	1 [Reference]	.006	120	65 (67.7)	1 [Reference]	.22	130	105 (66.9)	1 [Reference]	.008
Yes	121	83 (32.8)	0.68 (0.52-0.90)		57	31 (32.3)	0.75 (0.47-1.19)		64	52 (33.1)	0.62 (0.44-0.88)	

Abbreviations: ECOG, Eastern Cooperative Oncology Group; HR, hazard ratio; ICI, immune checkpoint inhibitor; MMR, mismatch repair; MSI-H, microsatellite instable; MSS, microsatellite stable; PPI, proton pump inhibitor; TTD, time to treatment discontinuation.

SI unit conversion: To convert albumin to g/L, multiply by 10.

^a^ Each patient had a diagnosis of metastatic colorectal cancer (*International Classification of Diseases, Ninth Revision [ICD-9]*: 153.x, 154.x; *ICD-10*: C18x, C19x, C20x, C21x) and at least 2 documented clinical visits between January 2013 and December 2019. Sensitivity analyses were carried out on patients diagnosed after Food and Drug Administration approval of ICIs for treatment of MSI-H metastatic colorectal cancer (May 2017).

^b^ Number of patients retained in the multivariate models.

^c^ Sample includes metastatic colorectal cancer patients who received immune checkpoint inhibitors between January 1, 2015, and December 31, 2019.

^d^ Sample includes patients with unknown MMR status.

^e^ Index date was the start of ICI-based therapy defined as the first administration or noncancelled order of any of the following drugs: nivolumab, pembrolizumab, atezolizumab, ipilimumab, tremelimumab, durvalumab, or avelumab.

^f^ For TTD, models were adjusted for sex, stage at initial diagnosis, primary cancer site, MMR status, *KRAS* mutation status, immunotherapy as first-line treatment, immunotherapy plus chemotherapy, albumin levels, and antibiotic use.

^g^ Immune checkpoint inhibitors given in combination with chemotherapeutic agents in the same line of therapy.

^h^ ECOG status and albumin levels were recorded 3 months before or after immunotherapy initiation.

^i^ Antibiotics or PPIs taken anytime from 1 month before the start of immunotherapy until the end.

For TTD, a significantly lower likelihood of immunotherapy discontinuation was observed for patients with MSI-H tumors (vs MSS cohort: HR, 0.41 [95% CI, 0.30-0.55]; *P* < .001), high albumin level (vs low: HR = 0.54, 95% CI, 0.39-0.76]; *P* < .001) and antibiotic use (vs nonuse: HR, 0.68 [95% CI, 0.52-0.90]; *P* = .006), while males (vs females: HR, 1.34 [95% CI, 1.04-1.73]; *P* = .02) and patients with synchronous mCRC (vs metachronous: HR, 1.65 [95% CI, 1.23-2.21]; *P* < .001) were significantly more likely to discontinue immunotherapy. In the MSI-H cohort, both sex (males vs females: HR, 1.67 [95% CI, 1.08-2.57]; *P* = .02) and combination therapy (vs monotherapy: HR, 2.02 [95% CI, 1.01-4.03]; *P* = .047) were associated with higher likelihood of immunotherapy discontinuation. On the other hand, patients with MSS tumors had a significant difference in immunotherapy discontinuation by disease stage (synchronous vs metachronous mCRC: HR, 1.92 [95% CI, 1.36-2.70]; *P* < .001), albumin level (high vs low: HR, 0.44 [95% CI, 0.29-0.67]; *P* < .001) and antibiotic use (yes vs no: HR, 0.62 [95% CI, 0.44-0.88]; *P* = .008). Of note, 29 out of 235 patients with MSS tumors (12.3%) had a durable response to ICIs as measured by a TTD greater than 6 months, while 16 out of the 122 patients with MSS/*KRAS*-mutated tumors (13.1%) had a durable response to ICIs.

When the analysis was restricted to patients diagnosed with mCRC after FDA approval, disease stage at initial diagnosis was no longer associated with either OS or TTD in the MSS cohort (eTable 9 in Supplement 1). In addition, sex was no longer significantly associated with either OS or TTD in the MSI-H cohort. Significant associations emerged between OS and rectal cancer (vs colon cancer: HR, 5.38 [95% CI, 1.04-27.76]; *P* = .045) or antibiotic use (vs nonuse: HR, 0.31 [95% CI, 0.1-0.99]; *P* = .048) in the MSI-H cohort.

### Sensitivity Analyses

A sensitivity analysis excluding patients with no structured activity within 90 days from mCRC diagnosis (n = 2638 [13.9%] of all patients; n = 55 [9.7%] of patients treated with immunotherapy) showed no substantial difference in associations in the multivariable models. In line with the main results, immunotherapy receipt was significantly lower in patients with synchronous mCRC (vs metachronous: OR, 0.60 [95% CI, 0.49-0.73]; *P* < .001), but significantly higher in patients with MSI-H tumors (vs MSS tumors: OR, 20.3 [95%CI = 16.1-25.7]; *P* < .001). In the MSI-H patient subgroup, immunotherapy was associated with a higher hazard of death among male patients (vs female patients: OR, 2.05 [95% CI, 1.15-3.63]; *P* = .01) and with a higher likelihood of immunotherapy discontinuation among patients with combination therapy (vs monotherapy: OR, 2.24 [95% CI, 1.12-4.45]; *P* = .02). However, unlike in the main analysis, patients with ECOG 2 to 4 had no survival benefits compared with patients with ECOG 0 to 1 (OR, 1.69 [95% CI, 0.86-3.33]; *P* = .13), while males had no higher likelihood of immunotherapy discontinuation compared with females (OR, 1.37 [95% CI, 0.91-2.08]; *P* = .13). In the MSS patient subgroup, we confirmed all associations observed in the main analysis; specifically, we observed associations between OS and disease stage at diagnosis (OR, 1.75 [95% CI, 1.15-2.65]; *P* = .008), albumin level (OR, 0.27 [95% CI, 0.17-0.43]; *P* < .001) and antibiotic use (OR, 0.43 [95% CI, 0.28-0.68]; *P* < .001), as well as associations between TTD and disease stage at diagnosis (OR, 1.74 [95% CI, 1.23-2.45]; *P* = .002), albumin level (OR, 0.42 [95% CI, 0.28-0.63]; *P* < .001), and antibiotic use (OR, 0.69 [95% CI, 0.48-0.99]; *P* = .04).

The multivariable logistic regression models and Cox proportional hazards models including missing values for MMR status, *BRAF* mutation status, *KRAS* mutation status, and ECOG status showed consistent associations compared with models without missing values for these variables. Patients who received immunotherapy were significantly less likely to have synchronous mCRC (vs metachronous: OR, 0.59 [95% CI, 0.49-0.71]; *P* < .001) but significantly more likely to carry MSI-H tumors (vs MSS tumors: OR, 0.71 [95% CI, 15.85-24.81]; *P* < .001). Among patients with MSI-H tumors treated with immunotherapy, the hazard of death was significantly higher among males (vs females: OR, 2.16 [95% CI, 1.25-3.72]; *P* = .006), but unlike the main results, ECOG 2 to 4 was not associated with reduced survival probability (vs 0-1: OR, 1.84 [95% CI, 0.97-3.48]; *P* = .06). On the other hand, the likelihood of immunotherapy discontinuation was no longer significantly higher in males (vs females: OR, 1.41 [95% CI, 0.95-2.1]; *P* = .09), but was still significantly higher in patients with combination therapy (vs monotherapy: OR, 2.13 [95% CI, 1.1-4.1]; *P* = .02). In the MSS patient subgroup treated with immunotherapy, we confirmed the associations between OS and disease stage at initial diagnosis (OR, 1.86 [95% CI, 1.25-2.77]; *P* = .002), albumin level (OR, 0.27 [95% CI, 0.17-0.43]; *P* < .001), and antibiotic use (OR, 0.41 [95% CI, 0.26-0.64]; *P* < .001), and the associations between TTD and disease stage at initial diagnosis (OR, 1.86 [95% CI, 1.35-2.58]; *P* < .001), albumin level (OR, 0.42 [95% CI, 0.28-0.63]; *P* < .001), and antibiotic use (OR, 0.63 [95% CI, 0.45-0.89]; *P* = .008).

## Discussion

In this study of routine clinical practice data from US oncology practices, patients with MSI-H mCRC who received ICIs in an early line of therapy had significantly longer survival expectancy and lower likelihood of therapy discontinuation compared with those treated with chemotherapy only. Our findings provide substantial evidence to support results from the Keynote 177 study toward higher efficacy of first-line pembrolizumab vs chemotherapy for patients with MSI-H mCRC.^15^ Although patients with MSS mCRC do not benefit from currently available ICI-based therapies, we found that 12.3% of patients with MSS tumors had durable responses following ICI-based therapy. We also found that antibiotics may confer an advantage to patients with MSS tumors treated with ICIs, which suggests a microbiome-mediated favorable modulation of the immune response.

Based on our analysis of factors associated with immunotherapy receipt, both MMR status and disease stage at CRC diagnosis emerged as potential key factors in ICI-based treatment decision-making regardless of pre- or post-FDA approval. In accordance with guidelines from earlier clinical trials,^2,3^ ICIs were most frequently prescribed as monotherapy (82.9%) and as secondary treatment following chemotherapy (77.4%).

Among patients with MSI-H tumors treated with immunotherapy, females and those with ECOG 0 to 1 (little to no impairment) had significantly higher survival probability than males and those with ECOG 2 to 4. Sex-based differences in survival have previously been reported in patients with mCRC and have been attributed to hormonal status as well as immunological factors.^16,17,18^ Tumors in women generally have lower antigenicity compared with men; however, in tumors with high mutational burden (such as MSI-H tumors), ICIs have been found to be more effective in women.^19^

Patients with MSI-H tumors who received ICIs as monotherapy had significantly lower likelihood of immunotherapy discontinuation than those who received combination therapy (immunotherapy plus chemotherapy in the same line of therapy). This finding could be secondary to lower toxic effects of immunotherapy and absence of chemotherapy-associated immunosuppression.^20^

Our findings provide routine clinical practice confirmation of the lack of substantial clinical benefits of ICI-based therapy for patients with MSS tumors with advanced CRC that has been reported in clinical trials.^21^ However, among patients with MSS mCRC treated with ICIs, synchronous disease was associated with increased hazard of death as well as higher likelihood of immunotherapy discontinuation than patients with metachronous disease. Further studies are needed to clarify the potential interaction between pathological stage and MMR status on survival outcomes among patients treated with immunotherapy.

High albumin level and antibiotic use were both associated with longer survival and reduced likelihood of immunotherapy discontinuation in the MSS cohort. High serum albumin level has previously been reported as a strong predictor of survival outcomes following ICI-based therapy in most cancer types,^22^ which could be related to the host’s ability to mount an effective immune response against the tumor.^23^ Our results differ from previous findings on concomitant exposure to ICIs and antibiotics in patients with cancer^24,25^ but are consistent with a previous study reporting an inverse association between antibiotic exposure and mortality in mCRC treated with chemotherapy.^26^ Antibiotics may act through modulation of the gut microbiome and differential priming of the immune repertoire, which has been shown to modulate response to ICIs in various cancer types.^27,28,29^ Alteration of the microbiome may be a potential strategy to enhance ICI-based treatment response, but more studies are needed to clarify the drug-microbiome interaction.

Despite the poor outcomes associated with synchronous disease among patients with MSS tumors, 12.3% of patients from the MSS cohort had a durable response to ICIs, showing that a subset of patients was, at least initially, responsive to ICI-based therapy. Durable responses to immunotherapy have been noted in patients with MSS tumors who harbor high tumor mutational burden or have proofreading mutations such as *POLE* mutations or upregulated PD-L1 expression.^30,31,32^ Factors affecting durable response in patients with MSS tumors warrant further investigation.

### Strengths and Limitations

Strengths of this study are the large and heterogeneous patient population, including patients underrepresented in clinical trials, and the use of EHR data from the Flatiron Health database with detailed treatment information. In addition, the sizeable MSS cohort that received ICI-based therapy (n = 235) gave us a unique opportunity to investigate factors associated with receipt and efficacy of ICIs in this understudied group.

This study also had limitations. These included incomplete or missing data in the EHRs such as receipt of surgery, comprehensive somatic genetic/molecular features, tumor mutational burden (TMB), drug-related adverse effects, and germline testing to determine hereditary or sporadic origin of the tumor.^33,34,35^

## Conclusions

In this cohort study of 18 932 patients with mCRC, the results supported findings from clinical trials about the benefits of ICIs as first-line treatment of patients with MSI-H mCRC. In addition, we provided important insights about characteristics of patients that may influence clinical outcomes in patients with mCRC treated with immunotherapy, especially for patients with MSS tumors that have generally been unresponsive to ICIs in clinical trials. Further research is needed to better understand the potential interaction between ICIs and patient/tumor characteristics and identify additional factors that may modulate the effect of ICIs on clinical outcomes.

## Notes

Supplement 1. **eMethods.** Supplementary Methods **eTable 1.** Antibiotic Use in Metastatic Colorectal Cancer Patients Treated With Immunotherapy **eTable 2.** Baseline Demographic and Clinical Characteristics of Patients With Metastatic Colorectal Cancer by Receipt of Immunotherapy **eTable 3.** Baseline Demographic and Clinical Characteristics of Metastatic Colorectal Cancer Patients by Receipt of Immunotherapy (Only Including Patients Diagnosed After May 2017) **eTable 4.** Multivariable Logistic Regression Models for Association Between Characteristics of Metastatic Colorectal Cancer Patients by Receipt of Immunotherapy (Only Including Patients Diagnosed After May 2017) **eTable 5.** Clinical Characteristics of Metastatic Colorectal Cancer Patients Treated With Immunotherapy **eTable 6.** Clinical Characteristics of Metastatic Colorectal Cancer Patients Treated With Immunotherapy (Only Including Patients Diagnosed After May 2017) **eTable 7.** Effect of No, Early (1st Line) or Late (2nd or Later Line) Treatment With Immununotherapy on Overall Survival in Patients With Metastatic Colorectal Cancer **eTable 8.** Univariate Models for Differences in OS and TTD Between Subgroups of Metastatic Colorectal Cancer Patients Treated With Immunotherapy **eTable 9.** Multivariable Cox Proportional Hazards Models for Differences in OS and TTD Among Metastatic Colorectal Cancer Patients Treated With Immunotherapy (Only Including Patients Diagnosed After May 2017) **eFigure.** Study Flow Diagram

Supplement 2.
Data Sharing Statement

## References

[1] Van Cutsem E, Cervantes A, Nordlinger B, Arnold D; ESMO Guidelines Working Group. Metastatic colorectal cancer: ESMO clinical practice guidelines for diagnosis, treatment and follow-up. Ann Oncol. 2014;25(suppl 3):iii1-iii9. doi:10.1093/annonc/mdu260 25190710

[2] Le DT, Kim TW, Van Cutsem E, . Phase II open-label study of pembrolizumab in treatment-refractory, microsatellite instability-high/mismatch repair-deficient metastatic colorectal cancer: KEYNOTE-164. J Clin Oncol. 2020;38(1):11-19. doi:10.1200/JCO.19.02107 31725351 PMC7031958

[3] Overman MJ, McDermott R, Leach JL, . Nivolumab in patients with metastatic DNA mismatch repair-deficient or microsatellite instability-high colorectal cancer (CheckMate 142): an open-label, multicentre, phase 2 study. Lancet Oncol. 2017;18(9):1182-1191. doi:10.1016/S1470-2045(17)30422-9 28734759 PMC6207072

[4] Overman MJ, Lonardi S, Wong KYM, . Durable clinical benefit with nivolumab plus ipilimumab in DNA mismatch repair-deficient/microsatellite instability-high metastatic colorectal cancer. J Clin Oncol. 2018;36(8):773-779. doi:10.1200/JCO.2017.76.9901 29355075

[5] André T, Lonardi S, Wong KYM, . Nivolumab plus low-dose ipilimumab in previously treated patients with microsatellite instability-high/mismatch repair-deficient metastatic colorectal cancer: 4-year follow-up from CheckMate 142. Ann Oncol. 2022;33(10):1052-1060. doi:10.1016/j.annonc.2022.06.008 35764271

[6] Smith KM, Desai J. Nivolumab for the treatment of colorectal cancer. Expert Rev Anticancer Ther. 2018;18(7):611-618. doi:10.1080/14737140.2018.1480942 29792730

[7] Casak SJ, Marcus L, Fashoyin-Aje L, . FDA approval summary: pembrolizumab for the first-line treatment of patients with MSI-H/dMMR advanced unresectable or metastatic colorectal carcinoma. Clin Cancer Res. 2021;27(17):4680-4684. doi:10.1158/1078-0432.CCR-21-0557 33846198 PMC8416693

[8] Kim HS, Lee S, Kim JH. Real-world evidence versus randomized controlled trial: clinical research based on electronic medical records. J Korean Med Sci. 2018;33(34):e213. doi:10.3346/jkms.2018.33.e213 30127705 PMC6097073

[9] Booth CM, Tannock IF. Randomised controlled trials and population-based observational research: partners in the evolution of medical evidence. Br J Cancer. 2014;110(3):551-555. doi:10.1038/bjc.2013.725 24495873 PMC3915111

[10] Blumenthal GM, Gong Y, Kehl K, . Analysis of time-to-treatment discontinuation of targeted therapy, immunotherapy, and chemotherapy in clinical trials of patients with non-small-cell lung cancer. Ann Oncol. 2019;30(5):830-838. doi:10.1093/annonc/mdz060 30796424

[11] Kemp R, Prasad V. Surrogate endpoints in oncology: when are they acceptable for regulatory and clinical decisions, and are they currently overused? BMC Med. 2017;15(1):134. doi:10.1186/s12916-017-0902-9 28728605 PMC5520356

[12] Flatiron Health. Flatiron Health database. Accessed September 1, 2022. https://flatiron.com/real-world-evidence/

[13] Zhang Q, Gossai A, Monroe S, Nussbaum NC, Parrinello CM. Validation analysis of a composite real-world mortality endpoint for patients with cancer in the United States. Health Serv Res. 2021;56(6):1281-1287. doi:10.1111/1475-6773.13669 33998685 PMC8586476

[14] Curtis MD, Griffith SD, Tucker M, . Development and validation of a high-quality composite real-world mortality endpoint. Health Serv Res. 2018;53(6):4460-4476. doi:10.1111/1475-6773.12872 29756355 PMC6232402

[15] Diaz LA Jr, Shiu KK, Kim TW, ; KEYNOTE-177 Investigators. Pembrolizumab versus chemotherapy for microsatellite instability-high or mismatch repair-deficient metastatic colorectal cancer (KEYNOTE-177): final analysis of a randomised, open-label, phase 3 study. Lancet Oncol. 2022;23(5):659-670. doi:10.1016/S1470-2045(22)00197-8 35427471 PMC9533375

[16] Hendifar A, Yang D, Lenz F, . Gender disparities in metastatic colorectal cancer survival. Clin Cancer Res. 2009;15(20):6391-6397. doi:10.1158/1078-0432.CCR-09-0877 19789331 PMC2779768

[17] Majek O, Gondos A, Jansen L, ; GEKID Cancer Survival Working Group. Sex differences in colorectal cancer survival: population-based analysis of 164,996 colorectal cancer patients in Germany. PLoS One. 2013;8(7):e68077. doi:10.1371/journal.pone.0068077 23861851 PMC3702575

[18] Klein SL, Flanagan KL. Sex differences in immune responses. Nat Rev Immunol. 2016;16(10):626-638. doi:10.1038/nri.2016.90 27546235

[19] Irelli A, Sirufo MM, D’Ugo C, Ginaldi L, De Martinis M. Sex and gender influences on cancer immunotherapy response. Biomedicines. 2020;8(7):232. doi:10.3390/biomedicines8070232 32708265 PMC7400663

[20] Xu C, Chen YP, Du XJ, . Comparative safety of immune checkpoint inhibitors in cancer: systematic review and network meta-analysis. BMJ. 2018;363:k4226. doi:10.1136/bmj.k4226 30409774 PMC6222274

[21] Le DT, Uram JN, Wang H, . PD-1 blockade in tumors with mismatch-repair deficiency. N Engl J Med. 2015;372(26):2509-2520. doi:10.1056/NEJMoa1500596 26028255 PMC4481136

[22] Yoo SK, Chowell D, Valero C, Morris LGT, Chan TA. Pre-treatment serum albumin and mutational burden as biomarkers of response to immune checkpoint blockade. NPJ Precis Oncol. 2022;6(1):23. doi:10.1038/s41698-022-00267-7 35393553 PMC8990074

[23] Takamizawa Y, Shida D, Boku N, . Nutritional and inflammatory measures predict survival of patients with stage IV colorectal cancer. BMC Cancer. 2020;20(1):1092. doi:10.1186/s12885-020-07560-3 33176752 PMC7656744

[24] Petrelli F, Iaculli A, Signorelli D, . Survival of Patients Treated with Antibiotics and Immunotherapy for Cancer: A Systematic Review and Meta-Analysis. J Clin Med. 2020;9(5):1458. doi:10.3390/jcm9051458 32414103 PMC7290584

[25] Serpas Higbie V, Rogers J, Hwang H, . Antibiotic exposure does not impact immune checkpoint blockade response in MSI-H/dMMR metastatic colorectal cancer: a single-center experience. Oncologist. 2022;27(11):952-957. doi:10.1093/oncolo/oyac162 35946836 PMC9632313

[26] Lu L, Zhuang T, Shao E, . Association of antibiotic exposure with the mortality in metastatic colorectal cancer patients treated with bevacizumab-containing chemotherapy: a hospital-based retrospective cohort study. PLoS One. 2019;14(9):e0221964. doi:10.1371/journal.pone.0221964 31504043 PMC6736303

[27] Vétizou M, Pitt JM, Daillère R, . Anticancer immunotherapy by CTLA-4 blockade relies on the gut microbiota. Science. 2015;350(6264):1079-1084. doi:10.1126/science.aad1329 26541610 PMC4721659

[28] Sivan A, Corrales L, Hubert N, . Commensal Bifidobacterium promotes antitumor immunity and facilitates anti-PD-L1 efficacy. Science. 2015;350(6264):1084-1089. doi:10.1126/science.aac4255 26541606 PMC4873287

[29] Gopalakrishnan V, Spencer CN, Nezi L, . Gut microbiome modulates response to anti-PD-1 immunotherapy in melanoma patients. Science. 2018;359(6371):97-103. doi:10.1126/science.aan4236 29097493 PMC5827966

[30] Wang C, Gong J, Tu TY, Lee PP, Fakih M. Immune profiling of microsatellite instability-high and polymerase ε (*POLE*)-mutated metastatic colorectal tumors identifies predictors of response to anti-PD-1 therapy. J Gastrointest Oncol. 2018;9(3):404-415. doi:10.21037/jgo.2018.01.09 29998005 PMC6006042

[31] Gong J, Robertson MD, Kim E, . Efficacy of PD-1 blockade in refractory microsatellite-stable colorectal cancer with high tumor mutation burden. Clin Colorectal Cancer. 2019;18(4):307-309. doi:10.1016/j.clcc.2019.08.001 31563511

[32] Fabrizio DA, George TJ Jr, Dunne RF, . Beyond microsatellite testing: assessment of tumor mutational burden identifies subsets of colorectal cancer who may respond to immune checkpoint inhibition. J Gastrointest Oncol. 2018;9(4):610-617. doi:10.21037/jgo.2018.05.06 30151257 PMC6087857

[33] Zlobec I, Kovac M, Erzberger P, . Combined analysis of specific KRAS mutation, BRAF and microsatellite instability identifies prognostic subgroups of sporadic and hereditary colorectal cancer. Int J Cancer. 2010;127(11):2569-2575. doi:10.1002/ijc.25265 20162668

[34] Cunningham JM, Kim CY, Christensen ER, . The frequency of hereditary defective mismatch repair in a prospective series of unselected colorectal carcinomas. Am J Hum Genet. 2001;69(4):780-790. doi:10.1086/323658 11524701 PMC1226064

[35] Kuismanen SA, Holmberg MT, Salovaara R, de la Chapelle A, Peltomäki P. Genetic and epigenetic modification of MLH1 accounts for a major share of microsatellite-unstable colorectal cancers. Am J Pathol. 2000;156(5):1773-1779. doi:10.1016/S0002-9440(10)65048-1 10793088 PMC1876911

