# Supplementary material for: Practice Patterns and Survival Outcomes of Immunotherapy for Metastatic Colorectal Cancer
Source: JAMA Netw Open. 2025 Mar 20;8(3):e251186. doi: 10.1001/jamanetworkopen.2025.1186 (PMC11926646; doi:10.1001/jamanetworkopen.2025.1186)
Supplement: Supplement 1. — eMethods. Supplementary Methods eTable 1. Antibiotic Use in Metastatic Colorectal Cancer Patients Treated With Immunotherapy eTable 2. Baseline Demographic and Clinical Characteristics of Patients With Metastatic Colorectal Cancer by Receipt of Immunotherapy eTable 3. Baseline Demographic and Clinical Characteristics of Metastatic Colorectal Cancer Patients by Receipt of Immunotherapy (Only Including Patients Diagnosed After May 2017) eTable 4. Multivariable Logistic Regression Models for Association Between Characteristics of Metastatic Colorectal Cancer Patients by Receipt of Immunotherapy (Only Including Patients Diagnosed After May 2017) eTable 5. Clinical Characteristics of Metastatic Colorectal Cancer Patients Treated With Immunotherapy eTable 6. Clinical Characteristics of Metastatic Colorectal Cancer Patients Treated With Immunotherapy (Only Including Patients Diagnosed After May 2017) eTable 7. Effect of No, Early (1st Line) or Late (2nd or Later Line) Treatment With Immununotherapy on Overall Survival in Patients With Metastatic Colorectal Cancer eTable 8. Univariate Models for Differences in OS and TTD Between Subgroups of Metastatic Colorectal Cancer Patients Treated With Immunotherapy eTable 9. Multivariable Cox Proportional Hazards Models for Differences in OS and TTD Among Metastatic Colorectal Cancer Patients Treated With Immunotherapy (Only Including Patients Diagnosed After May 2017) eFigure. Study Flow Diagram [file jamanetwopen-e251186-s001.pdf]

Supplemental Online Content

Bari S, Matejcic M, Kim RD, et al. Practice patterns and survival outcomes of immunotherapy for metastatic colorectal cancer. *JAMA Netw Open.* 2025;8(3):e251186. doi:10.1001/jamanetworkopen.2025.1186

eMethods. Supplementary Methods

eTable 1. Antibiotic Use in Metastatic Colorectal Cancer Patients Treated With Immunotherapy

eTable 2. Baseline Demographic and Clinical Characteristics of Patients With Metastatic Colorectal Cancer by Receipt of Immunotherapy

eTable 3. Baseline Demographic and Clinical Characteristics of Metastatic Colorectal Cancer Patients by Receipt of Immunotherapy (Only Including Patients Diagnosed After May 2017)

eTable 4. Multivariable Logistic Regression Models for Association Between Characteristics of Metastatic Colorectal Cancer Patients by Receipt of Immunotherapy (Only Including Patients Diagnosed After May 2017)

eTable 5. Clinical Characteristics of Metastatic Colorectal Cancer Patients Treated With Immunotherapy

eTable 6. Clinical Characteristics of Metastatic Colorectal Cancer Patients Treated With Immunotherapy (Only Including Patients Diagnosed After May 2017)

eTable 7. Effect of No, Early (1st Line) or Late (2nd or Later Line) Treatment With Immununotherapy on Overall Survival in Patients With Metastatic Colorectal Cancer

eTable 8. Univariate Models for Differences in OS and TTD Between Subgroups of Metastatic Colorectal Cancer Patients Treated With Immunotherapy

eTable 9. Multivariable Cox Proportional Hazards Models for Differences in OS and TTD Among Metastatic Colorectal Cancer Patients Treated With Immunotherapy (Only Including Patients Diagnosed After May 2017)

eFigure. Study Flow Diagram

This supplemental material has been provided by the authors to give readers additional information about their work.

© 2025 Bari S et al. *JAMA Network Open*.

eMethods. Supplementary Material

Data source and patient selection

The Flatiron Health database is a longitudinal database, comprising de-identified patient-level structured and unstructured data, curated via technology-enabled abstraction [1,2]. The de-identified data originated from approximately 280 US cancer clinics (~800 sites of care) [3]. Structured data (e.g. demographics, diagnosis codes, laboratory results, medication administrations) were harmonized across different source systems to standard reference terminologies. Unstructured data (e.g. treatment history, radiology/pathology reports, discharge notes) were curated via technology-enabled chart abstraction from physicians' notes supplemented by manual review, as previously described [4].

The majority of patients in the database originate from community oncology settings; relative community/academic proportions may vary depending on study cohort. Eligibility requirements included age 18 years or older, diagnosis of pathologically confirmed metastatic colorectal cancer (mCRC), and at least one line of systemic therapy on or after the date of diagnosis. The diagnosis was surfaced from the International Classification of Diseases 9th Revision (ICD-9)/10th Revision (ICD-10) codes (ICD-9: 153.x, 154.x; ICD-10: C18x, C19x, C20x, C21x) [5,6] and confirmed by abstraction. Of the 23,963 patients with available data, 5,031 were excluded for any of the following reasons: (i) missing visit, drug administration or non-canceled order after metastatic diagnosis (n=225), (ii) missing regimen or line of therapy information (n=4,795), (iii) undetermined start or end of treatment date (n=4), (iv) incomplete diagnosis information (n=5), and (v) death or censoring date before start of treatment date (n=2). The final study population included 18,932 mCRC patients.

Index date definition and censoring

In the overall sample analyses (n=18,932), index date was defined as the first episode of any systemic treatment regimen administered on or after the date of metastatic disease being diagnosed (Figure 1, eTable 6). In analyses conducted exclusively among patients treated with immunotherapy (n=566), index date was the start of ICI-based therapy defined as the first administration or non-cancelled order of any of the following drugs: nivolumab, pembrolizumab, atezolizumab, ipilimumab, tremelimumab, durvalumab, or avelumab (Table 3, Figure 2, eTable 7, eTable 8).

Medication, mortality and visit information were used to select censored patients. For overall survival (OS) analysis, patients were censored at their last administration date if none of the following criteria were met: (i) the patient advanced to a subsequent line of therapy, (ii) had a recorded date of death, or (iii) the gap between the last drug administration or non-canceled order and confirmed structured activity was <120 days. The 120-day gap rule was used to select patients who were likely to have missing treatment data. For time to treatment discontinuation (TTD) analysis, patients were considered as having discontinued immunotherapy if any of the following occurred: (i) the patient started a subsequent non-ICI systemic therapy after the ICI regimen, (ii) the patient died, or (iii) there was a >120-day gap between the patient's last drug administration or non-canceled order and confirmed structured activity. If none of the discontinuation criteria were met, patients were treated as censored at their last known ICI administration date. Patients initiating systemic therapy after June 2019 were censored to allow at least six months of follow-up after diagnosis.

Data extraction

Patient-level data from January 1, 2013, through December 31, 2019, were utilized. Information on systemic treatment, as evidenced by order and administration dates of antineoplastic agents recorded in the EHR, was used to establish oncologist-defined, rule-based lines of therapy [7]. In our study, "disease stage at initial diagnosis" discerned patients diagnosed with stage IV disease at initial diagnosis (synchronous mCRC) versus those twho were initially diagnosed with stage I-III disease but later developed metastatic disease (metachronous mCRC). Patients were stratified into three groups based on when immunotherapy was first administered (first, second, third or later line of therapy). We also defined the "immunotherapy plus chemotherapy" variable to classify patients based on whether immune checkpoint inhibitors were administered as monotherapy or in combination with chemotherapeutic agents within a line of therapy. Microsatellite instable-high (MSI-H) status of tumor samples was assessed through a combination of Mismatch Repair (MMR) immunohistochemistry, MSI polymerase chain reaction, and MSI next-generation sequencing. *KRAS*, *BRAF* and *NRAS* gene mutational status were abstracted from clinical test results. Eastern Cooperative Oncology Group (ECOG) performance status and blood albumin level were recorded within three months prior to or after immunotherapy initiation. ECOG score was categorized as 0-1 (little to no impairment) and 2-4 (some to high impairment). Albumin level was categorized as ≤3 g/dl (low) and >3 g/dl (high), to evaluate the effect of severe protein malnutrition. Antibiotic and proton pump inhibitor (PPI) use was recorded from one month before until the end of immunotherapy.

© 2025 Bari S et al. *JAMA Network Open*.

Sensitivity analysis

The primary analyses were restricted to patients diagnosed with mCRC after Food and Drug Administration (FDA) approval of ICIs for treatment of refractory MSI-H mCRC. We also excluded patients with >90-day gap between date of diagnosis of mCRC and date of first structured activity (n=2,638), as these patients may have received therapy outside of the Flatiron Health network. To maintain sufficient sample size, missing values for MMR status (38.4%), *BRAF* mutation status (50.6%), *KRAS* mutation status (22.5%), and ECOG status (16.3%) were recoded as an additional category for each respective variable in the multivariable models.

References

1. Ma X, Long L, Moon S, Adamson BJS, Baxi SS. *Comparison of Population Characteristics in Real-World Clinical Oncology Databases in the US: Flatiron Health, SEER, and NPCR*. Oncology; 2020. doi:10.1101/2020.03.16.20037143
2. Birnbaum B, Nussbaum N, Seidl-Rathkopf K, et al. Model-assisted cohort selection with bias analysis for generating large-scale cohorts from the EHR for oncology research. Published online January 13, 2020. Accessed May 16, 2023. http://arxiv.org/abs/2001.09765
3. Flatiron Health. Flatiron Health database. Accessed September 1, 2022. https://flatiron.com/real-world-evidence/
4. Abernethy AP, Gippetti J, Parulkar R, Revol C. Use of Electronic Health Record Data for Quality Reporting. *J Oncol Pract*. 2017;13(8):530-534. doi:10.1200/JOP.2017.024224
5. International Classification of Diseases , ninth revision (ICD-9). Centers for Disease Control and Prevention. National Center for Health Statistics. Accessed October 27, 2022. https://www.cdc.gov/nchs/icd/icd9.htm
6. International Classification of Diseases, tenth revision (ICD-10). Centers for Disease Control and Prevention. National Center for Health Statistics. Accessed October 27, 2022. https://www.cdc.gov/nchs/icd/icd10.htm
7. Khozin S, Abernethy AP, Nussbaum NC, et al. Characteristics of Real-World Metastatic Non-Small Cell Lung Cancer Patients Treated with Nivolumab and Pembrolizumab During the Year Following Approval. *Oncologist*. 2018;23(3):328-336. doi:10.1634/theoncologist.2017-0353

© 2025 Bari S et al. *JAMA Network Open*.

Supplementary Tables

eTable 1. Antibiotic use in metastatic colorectal cancer patients treated with immunotherapy

Antibiotic classes, n (%)	Individual antibiotics [2]	Patients (n=566) [3,4,5]	MMR status [1]	
			MSI-H (n=234)	MSS (n=235)
Antifungals	fluconazole, ketoconazole, fluconazole in sodium chloride, caspofungin acetate, voriconazole, fluconazole in dextrose, anidulafungin.	19 (3.4)	6 (2.6)	9 (3.8)
Antivirals	valacyclovir hcl, acyclovir, famciclovir, oseltamivir phosphate, acyclovir sodium, valganciclovir hcl, ganciclovir sodium.	13 (2.3)	6 (2.6)	4 (1.7)
Vancomycin	vancomycin hcl, vancomycin in 0.9 % sodium chloride, vancomycin in 5 % dextrose in water.	7 (1.2)	6 (2.6)	0 (0)
Antianaerobics	metronidazole, amoxicillin/potassium clavulanate, clindamycin hcl, ertapenem sodium, metronidazole in sodium chloride, ampicillin sodium/sulbactam sodium, piperacillin sodium/tazobactam sodium, piperacillin/tazobactam in dextrose, meropenem, ticarcillin disodium/potassium clavulanate.	32 (5.7)	16 (6.8)	14 (6.0)
Gram-negative	cephalexin, cefpodoxime proxetil, ceftriaxone sodium, cefuroxime axetil, cefdinir, cefepime, cefazolin sodium, ceftazidime, cefoxitin sodium, cefotetan sodium, "cefotaxime sodium, cefuroxime sodium, cefazolin sodium/dextrose, ceftaroline fosamil acetate.	24 (4.2)	8 (3.4)	8 (3.4)
Gram-positive	vancomycin hcl, linezolid, erythromycin base, vancomycin in 0.9 % sodium chloride, erythromycin lactobionate, dicloxacillin sodium, daptomycin, oxacillin sodium, linezolid in 5% dextrose-water, penicillin g potassium, penicillin g potassium/dextrose-water, vancomycin in 5 % dextrose-water.	7 (1.2)	6 (2.6)	0 (0)
Broad-spectrum antibiotics	azithromycin, ciprofloxacin hcl, sulfamethoxazole/trimethoprim, amoxicillin/potassium clavulanate, minocycline hcl, doxycycline hyclate, ciprofloxacin/ciprofloxacin hcl, doxycycline monohydrate, ertapenem sodium, moxifloxacin hcl, gentamicin sulfate, doxycycline monohydrate/salicylic acid/octinoxate/zinc oxide, erythromycin base, doxycycline monohydrate/benzoyl peroxide, ciprofloxacin lactate in 5% dextrose-water, ampicillin sodium/sulbactam sodium, piperacillin and tazobactam in dextrose, piperacillin sodium/tazobactam sodium, ampicillin sodium, doxycycline calcium, neomycin sulfate", "erythromycin lactobionate, meropenem, gentamicin sulfate, moxifloxacin hcl in sodium chloride, tobramycin sulfate, ticarcillin disodium/potassium clavulanate, doxycycline hyclate, gentamicin sulfate in sodium chloride, gentamicin sulfate/sodium chloride.	102 (18.0)	45 (19.2)	39 (16.6)

[1] Patients with unknown MMR status (n=97) were excluded
[2] Only antibiotics used by patients who received immune checkpoint inhibitors (n=566) are listed
[3] Number of patients taking antibiotics anytime from one month before the start of immunotherapy until the end
[4] Sample includes metastatic colorectal cancer patients who received immune checkpoint inhibitors between January 1, 2015 and December 31, 2019
[5] Including patients with unknown MMR status (n=97)
Abbreviations:
MMR = mismatch repair
MSI-H = microsatellite instable-high
MSS = microsatellite stable

© 2025 Bari S et al. *JAMA Network Open*.

eTable 2. Baseline demographic and clinical characteristics of metastatic colorectal cancer patients by receipt of immunotherapy

		Immunotherapy receipt		
	All patients, n (%)[1]	No, n (%)	Yes, n (%)	P-value [2]
Total	18932	18366	566	
Characteristics				
Demographic characteristics				
**Sex**				.009
Female	8394 (44.3)	8112 (44.2)	282 (49.8)	
Male	10537 (55.7)	10253 (55.8)	284 (50.2)	
Unknown [3]	1 (0)	1 (0)	0 (0)	
**Race**				.28
Asian	546 (2.9)	533 (2.9)	13 (2.3)	
Black or African American	2005 (10.6)	1952 (10.6)	53 (9.3)	
White	12338 (65.2)	11946 (65.0)	392 (69.3)	
Unknown	4043 (21.4)	3935 (21.4)	108 (19.1)	
**Ethnicity**				.26
Hispanic/Latino	1674 (8.8)	1632 (8.9)	42 (7.4)	
Non-Hispanic/non-Latino	17258 (91.2)	16734 (91.1)	524 (92.6)	
**Insured at initial diagnosis**				.045
No	6016 (31.8)	5806 (31.6)	210 (37.1)	
Yes	10300 (54.4)	10000 (54.4)	300 (53.0)	
Unknown	2616 (13.8)	2560 (13.9)	56 (9.9)	
Clinical characteristics				
Median age at metastatic diagnosis, y (Q1-Q3)	64.6 (55.0-73.3)	64.6 (55.0-73.3)	64.4 (52.9-73.4)	.27
**Practice type**				.94
Academic	1100 (5.8)	1068 (5.8)	32 (5.7)	
Community	17832 (94.2)	17298 (94.2)	534 (94.3)	
**Disease stage at initial diagnosis** [4]				<.001
I-III	6791 (35.9)	6505 (35.4)	286 (50.5)	
IV	11451 (60.5)	11200 (61.0)	251 (44.3)	
Unknown	690 (3.6)	661 (3.6)	29 (5.1)	
**Primary cancer site**				<.001
Colon	14023 (74.1)	13564 (73.9)	459 (81.1)	
Rectum	4534 (23.9)	4433 (24.1)	101 (17.8)	
Colorectal NOS	375 (2.0)	369 (2.0)	6 (1.1)	
**Disease diagnosis after FDA approval** [5]				.02
No	12481 (65.9)	12135 (66.1)	346 (61.1)	
Yes	6451 (34.1)	6231 (33.9)	220 (38.9)	
Tumor characteristics				
**MMR status**				<.001
MSI-H	726 (3.8)	492 (2.7)	234 (41.3)	

© 2025 Bari S et al. *JAMA Network Open*.

MSS	10927 (57.7)	10692 (58.2)	235 (41.5)	
Unknown	7279 (38.4)	7182 (39.1)	97 (17.1)	
***KRAS* mutation status**				.55
Wild-type	7858 (41.5)	7593 (41.3)	265 (46.8)	
Positive	6823 (36.0)	6606 (36.0)	217 (38.3)	
Unknown	4251 (22.5)	4167 (22.7)	84 (14.8)	
***BRAF* mutation status**				<.001
Wild-type	8339 (44.1)	8053 (43.8)	286 (50.5)	
Positive	1012 (5.3)	913 (5.0)	99 (17.5)	
Unknown	9581 (50.6)	9400 (51.2)	181 (32.0)	
***NRAS* mutation status**				.45
Wild-type	8466 (44.7)	8127 (44.3)	339 (59.9)	
Positive	470 (2.5)	455 (2.5)	15 (2.7)	
Unknown	9996 (52.8)	9784 (53.3)	212 (37.4)	

[1] Sample includes patients diagnosed with metastatic colorectal cancer between January 1, 2015 and December 31, 2019
[2] P-value for difference by immunotherapy receipt (yes vs no): Kruskal-Wallis for continuous variables; chi-square test for categorical variables
[3] The missing category is not shown for variables with no missing information
[4] All patients in the cohort were either diagnosed with metastatic colorectal cancer or had progressed to metastatic disease after early-stage diagnosis
[5] Date of of immune checkpoint inhibitors for treatment of MSI-H metastatic colorectal cancer (May 2017)
Abbreviations:
Colorectal NOS = colorectal cancer not otherwise specified
MMR = mismatch repair
MSI-H = microsatellite instable-high
MSS = microsatellite stable

© 2025 Bari S et al. *JAMA Network Open*.

eTable 3. Baseline demographic and clinical characteristics of metastatic colorectal cancer patients by receipt of immunotherapy (only including patients diagnosed after May 2017)

		Immunotherapy receipt [1]		
	All patients, n (%)	No, n (%)	Yes, n (%)	P-value [2]
Total	6451	6231	220	
Characteristics				
Demographic characteristics				
**Sex**				0.0053
Female	2854 (44.2)	2736 (43.9)	118 (53.6)	
Male	3597 (55.8)	3495 (56.1)	102 (46.2)	
**Race**				0.66
Asian	201 (3.1)	194 (3.1)	7 (3.2)	
Black or African American	678 (10.5)	657 (10.5)	21 (9.5)	
White	3944 (61.1)	3794 (60.9)	150 (68.2)	
Unknown [3]	1628 (25.2)	1586 (25.5)	42 (19.1)	
**Ethnicity**				0.034
Hispanic/Latino	564 (8.7)	554 (8.9)	10 (4.5)	
Non-Hispanic/non-Latino	5887 (91.3)	5677 (91.1)	210 (95.5)	
**Insured at initial diagnosis**				0.582
No	1724 (26.7)	1660 (26.6)	64 (29.1)	
Yes	3994 (61.9)	3859 (61.9)	135 (61.4)	
Unknown	733 (11.4)	712 (11.4)	21 (9.5)	
Clinical characteristics				
Median age at metastatic diagnosis, y (Q1-Q3)	64.42 (54.8-73.1)	64.32 (54.8-73.0)	66.95 (55.0-75.4)	0.049
**Practice type**				0.33
Academic	343 (5.3)	335 (5.4)	8 (3.6)	
Community	6108 (94.7)	5896 (94.6)	212 (96.4)	
**Disease stage at initial diagnosis** [4]				$9.23x10^{-14}$
I-III	2269 (35.2)	2142 (34.4)	127 (57.7)	
IV	4003 (62.1)	3921 (62.9)	82 (37.3)	
Unknown	179 (2.8)	168 (2.7)	11 (5.0)	
**Primary cancer site**				$2.48x10^{-05}$
Colon	4784 (74.2)	4594 (73.7)	190 (86.4)	
Rectum	1542 (23.9)	1516 (24.3)	26 (11.8)	
Colorectal NOS	125 (1.9)	121 (1.9)	4 (1.8)	
Tumor characteristics				
**MMR status**				$<2.2x10^{-16}$
MSI-H	319 (4.9)	177 (2.8)	142 (64.5)	
MSS	4899 (75.9)	4844 (77.7)	55 (25.0)	
Unknown	1233 (19.1)	1210 (19.4)	23 (10.5)	
***KRAS* mutation status**				$5.58x10^{-05}$
Wild-type	2569 (39.8)	2453 (39.4)	116 (52.7)	

© 2025 Bari S et al. *JAMA Network Open*.

Positive	2398 (37.2)	2341 (37.6)	57 (25.9)	
Unknown	1484 (23.0)	1437 (23.1)	47 (21.4)	
***BRAF* mutation status**				$<2.2x10^{-16}$
Wild-type	3730 (57.8)	3621 (58.1)	109 (49.5)	
Positive	433 (6.7)	378 (6.1)	55 (25.0)	
Unknown	2288 (35.5)	2232 (35.8)	56 (25.5)	
***NRAS* mutation status**				0.093
Wild-type	3800 (58.9)	3650 (58.6)	150 (68.2)	
Positive	211 (3.3)	208 (3.3)	3 (1.4)	
Unknown	2440 (37.8)	2373 (38.1)	67 (30.5)	

[1] Sample includes patients diagnosed after FDA approval of immune checkpoint inhibitors for treatment of MSI-H metastatic colorectal cancer (May 2017)
[2] P-value for difference by immunotherapy receipt (yes vs no): Kruskal-Wallis for continuous variables; chi-square test for categorical variables
[3] The missing category is not shown for variables with no missing information
[4] All patients in the cohort were either diagnosed with metastatic colorectal cancer or had progressed to metastatic disease after early-stage diagnosis
Abbreviations:
Colorectal NOS = colorectal cancer not otherwise specified
MMR = mismatch repair
MSI-H = microsatellite instable-high
MSS = microsatellite stable

© 2025 Bari S et al. *JAMA Network Open*.

eTable 4. Multivariable logistic regression models for association between characteristics of metastatic colorectal cancer patients by receipt of immunotherapy (only including patients diagnosed after May 2017)

	All patients (n=3696) [1,2,3]			MSI-H (n=253)			MSS (n=3443)		
Characteristics	Immunotherapy (no/yes)	Adj OR (95% CI) [4]	P-value	Immunotherapy (no/yes)	Adj OR (95% CI)	P-value	Immunotherapy (no/yes)	Adj OR (95% CI)	P-value
**Sex**			0.52			0.63			0.77
Female	1559/79	1 (Ref)		67/63	1 (Ref)		1492/16	1 (Ref)	
Male	1987/71	0.87 (0.57; 1.33)		71/52	0.87 (0.5; 1.52)		1916/19	0.9 (0.46; 1.77)	
**Disease stage at initial diagnosis** [5]			$2.4x10^{-06}$			$1.9x10^{-05}$			0.032
I-III	1301/95	1 (Ref)		53/76	1 (Ref)		1248/19	1 (Ref)	
IV	2245/55	0.37 (0.25; 0.56)		85/39	0.32 (0.19; 0.54)		2160/16	0.48 (0.24; 0.94)	
**Primary cancer site**			0.65			0.92			0.66
Colon	2724/136	1 (Ref)		129/109	1 (Ref)		2595/27	1 (Ref)	
Rectum	822/14	0.86 (0.45; 1.64)		9/6	0.94 (0.31; 2.9)		813/8	0.84 (0.37; 1.86)	
**MMR status**			$1.3x10^{-75}$			–			–
MSS	3408/35	1 (Ref)		–	1 (Ref)		–	1 (Ref)	
MSI-H	138/115	71.62 (45.44; 112.89)		–	–		–	–	
***BRAF* mutation status**			0.51			0.31			0.49
Wild-type	3192/96	1 (Ref)		88/63	1 (Ref)		3104/33	1 (Ref)	
Positive	354/54	1.18 (0.72; 1.93)		50/52	1.34 (0.76; 2.36)		304/2	0.6 (0.14; 2.55)	

[1] Number of patients retained in the multivariate models
[2] Sample includes patients diagnosed after FDA approval of immune checkpoint inhibitors for treatment of MSI-H metastatic colorectal cancer (May 2017)
[3] Sample includes patients with unknown MMR status
[4] Models were adjusted for gender, stage at initial diagnosis, primary cancer site, MMR status, and BRAF mutation status
[5] All patients in the cohort were either diagnosed with metastatic colorectal cancer or had progressed to metastatic disease after early-stage diagnosis

Abbreviations:
OR = hazard ratio
CI = confidence interval
MMR = mismatch repair
MSI-H = microsatellite instable-high
MSS = microsatellite stable

© 2025 Bari S et al. *JAMA Network Open*.

eTable 5. Clinical characteristics of metastatic colorectal cancer patients treated with immunotherapy

		MMR status [1]		
	All patients, n (%) [2,3]	MSI-H, n (%)	MSS, n (%)	P-value [4]
Total	566	234	235	
Characteristics				
Median age at metastatic diagnosis, y (Q1-Q3)	64.4 (52.9-73.4)	66.5 (53.5-76.0)	60.4 (52.5-69.7)	6.9×10^{-04}
**Disease stage at initial diagnosis** [5]				0.017
I-III	286 (50.5)	135 (57.7)	45 (46.4)	
IV	251 (44.3)	92 (39.3)	43 (44.3)	
Unknown [6]	29 (5.1)	7 (3.0)	9 (9.3)	
**Immunotherapy as first treatment**				1.9×10^{-08}
No	438 (77.4)	154 (65.8)	207 (88.1)	
Yes	128 (22.6)	80 (34.2)	28 (11.9)	
**Immunotherapy plus chemotherapy** [7]				1.9×10^{-08}
No	469 (82.9)	219 (93.6)	174 (74.0)	
Yes	97 (17.1)	15 (6.4)	61 (26.0)	
**ECOG status** [8]				1
0-1	356 (62.9)	148 (63.2)	149 (63.4)	
2-4	94 (16.6)	39 (16.7)	39 (16.6)	
Not eligible [9]	24 (4.2)	10 (4.3)	12 (5.1)	
Unknown	92 (16.3)	37 (15.8)	35 (14.9)	
**Albumin levels** [8]				0.88
<3 g/dL	83 (14.7)	32 (13.7)	36 (15.3)	
≥3 g/dL	456 (80.6)	186 (79.5)	191 (81.3)	
Not eligible [9]	11 (1.9)	8 (3.4)	6 (2.6)	
Unknown	16 (2.8)	8 (3.4)	2 (0.8)	
**Antibiotic use** [10]				1
No	389 (68.7)	160 (68.4)	160 (68.1)	
Yes	177 (31.3)	74 (31.6)	75 (31.9)	
**PPI use, n (%)** [10]				0.12
No	484 (85.5)	205 (87.6)	195 (83.0)	
Yes	82 (14.5)	29 (12.4)	40 (17.0)	
**Immunotherapy regimen** [11]				NA
Atezolizumab	22 (3.5)	1 (0.4)	12 (4.5)	
Atezolizumab / Ipilimumab	1 (0.2)	0 (0)	1 (0.4)	
Durvalumab	1 (0.2)	0 (0)	0 (0)	
Ipilimumab / Nivolumab	34 (5.4)	21 (8.4)	10 (3.7)	
Nivolumab	218 (34.8)	64 (25.7)	117 (43.8)	
Pembrolizumab	351 (56.0)	163 (65.5)	127 (47.6)	

[1] Patients with unknown MMR status were excluded
[2] Sample includes metastatic colorectal cancer patients who received immune checkpoint inhibitors between January 1, 2015 and December 31, 2019

© 2025 Bari S et al. *JAMA Network Open*.

[3] Sample includes patients with unknown MMR status
[4] P-value for difference by MMR status (MSI-H vs MSS): Kruskal-Wallis for continuous variables; chi-square test for categorical variables; p-value not presented for variables with more than two categories
[5] All patients in the cohort were either diagnosed with metastatic colorectal cancer or had progressed to metastatic disease after early-stage diagnosis
[6] The missing category is not shown for variables with no missing information
[7] Immune checkpoint inhibitors given in combination with chemotherapeutic agents in the same line of therapy
[8] ECOG status and albumin levels were recorded 3 months before or after immunotherapy initiation
[9] Patients not eligible because measurements was taken >3 months before or after immunotherapy initiation
[10] Antibiotics or PPIs taken anytime from one month before the start of immunotherapy until the end
[11] If one patient used the same drug in two distinct lines of therapy, it would be counted twice (one for each line of therapy)
Abbreviations:
MMR = mismatch repair
MSI-H = microsatellite instable-high
MSS = microsatellite stable
ECOG = Eastern Cooperative Oncology Group
PPI = proton pump inhibitor

© 2025 Bari S et al. *JAMA Network Open*.

eTable 6. Clinical characteristics of metastatic colorectal cancer patients treated with immunotherapy (only including patients diagnosed after May 2017)

		MMR status [1]		
	All patients, n (%) [2,3]	MSI-H, n (%)	MSS, n (%)	P-value [4]
Total	220	142	55	
Characteristics				
Median age at metastatic diagnosis, y (Q1-Q3)	67.0 (55.0-75.4)	68.3 (55.7-78.0)	63.7 (53.4-74.0)	0.099
**Disease stage at initial diagnosis)** [5]				0.33
I-III	127 (57.7)	89 (62.7)	28 (50.9)	
IV	82 (37.3)	50 (35.2)	23 (41.8)	
Unknown [6]	11 (5.0)	3 (2.1)	4 (7.3)	
**Immunotherapy as first treatment**				0.0011
No	131 (59.5)	73 (51.4)	43 (78.2)	
Yes	89 (40.5)	69 (48.6)	12 (21.8)	
**Immunotherapy plus chemotherapy** [7]				$1.063x10^{-05}$
No	187 (85.0)	132 (93.0)	37 (67.3)	
Yes	33 (15.0)	10 (7.0)	18 (32.7)	
**ECOG status** [8]				0.96
0-1	142 (64.5)	88 (62.0)	37 (67.3)	
2-4	43 (19.5)	29 (20.4)	11 (20.0)	
Not eligible [9]	7 (3.2)	3 (2.1)	2 (3.6)	
Unknown	28 (12.7)	20 (14.1)	5 (9.1)	
**Albumin levels** [8]				0.74
<3 g/dL	32 (14.5)	22 (15.5)	7 (12.7)	
≥3 g/dL	176 (80.0)	111 (78.2)	47 (85.5)	
Not eligible [9]	5 (2.3)	5 (3.5)	0 (0)	
Unknown	7 (3.2)	4 (2.8)	1 (1.8)	
**Antibiotic use** [10]				0.92
No	162 (73.6)	103 (72.5)	41 (74.5)	
Yes	58 (26.4)	39 (27.5)	14 (25.5)	
**PPI use** [10]				0.13
No	197 (89.5)	129 (90.8)	45 (81.8)	
Yes	23 (10.5)	13 (9.2)	10 (18.2)	
**Immunotherapy regimen** [11]				NA
Atezolizumab	5 (2.1)	1 (0.7)	1 (1.6)	
Atezolizumab / Ipilimumab	1 (0.4)	0 (0)	1 (1.6)	
Durvalumab	1 (0.4)	0 (0)	0 (0)	
Ipilimumab / Nivolumab	24 (10.0)	19 (12.6)	3 (4.9)	
Nivolumab	71 (29.7)	40 (26.5)	23 (37.7)	
Pembrolizumab	137 (57.3)	91 (60.3)	33 (54.1)	

[1] Patients with unknown MMR status were excluded

[2] Sample includes patients diagnosed after FDA approval of immune checkpoint inhibitors for treatment of MSI-H metastatic colorectal cancer (May 2017)

© 2025 Bari S et al. *JAMA Network Open*.

[3] Sample includes patients with unknown MMR status
[4] P-value for difference by MMR status (MSI-H vs MSS): Kruskal-Wallis for continuous variables; chi-square test for categorical variables; p-value not presented for variables with more than two categories
[5] All patients in the cohort were either diagnosed with metastatic colorectal cancer or had progressed to metastatic disease after early-stage diagnosis
[6] The missing category is not shown for variables with no missing information
[7] Immune checkpoint inhibitors given in combination with chemotherapeutic agents in the same line of therapy
[8] ECOG status and albumin levels were recorded 3 months before or after immunotherapy initiation
[9] Patients not eligible because measurements was taken >3 months before or after immunotherapy initiation
[10] Antibiotics or PPIs taken from one month before the start of immunotherapy until the end
[11] If one patient used the same drug in two distinct lines of therapy, it would be counted twice (one for each line of therapy)

Abbreviations:

- MMR = mismatch repair
- MSI-H = microsatellite instable-high
- MSS = microsatellite stable
- ECOG = Eastern Cooperative Oncology Group
- PPI = proton pump inhibitor

© 2025 Bari S et al. *JAMA Network Open*.

eTable 7. Effect of no, early (1st line) or late (2nd or later line) treatment with immununotherapy on overall survival in patients with metastatic colorectal cancer

	Patients, n	Events, n	Median (95%CI), mo	Log-rank p-value	Adj HR (95%CI), p-value
**OS**					
**All patients (n=6,677) [1,2,3]**					
	Patients, n	Events, n [4]	Median OS (95%CI), mo	Log-rank p-value	Adj HR (95%CI), p-value [5]
no immunotherapy	6,409	3,142	20.7 (20.3-21.1)	$2x10^{-10}$	Ref
immunotherapy at 1st line [6]	137	47	30.2 (19.9-NA)		0.60 (0.44; 0.82), 0.0015
immunotherapy at 2nd or later line [6]	131	90	36.1 (31.5-40.1)		0.85 (0.69; 1.05), 0.13
**MSS tumor carriers (n=6,283)**					
	Patients, n	Events, n	Median OS (95%CI), mo	Log-rank p-value	Adj HR (95%CI), p-value
no immunotherapy	6,148	2,992	25.3 (24.7-26.1)	0.009	Ref
immunotherapy at 1st line	35	15	18.9 (12.1-NA)		1.30 (0.78; 2.15), 0.32
immunotherapy at 2nd or later line	100	74	35.0 (30.0-41.1)		0.89 (0.71; 1.12), 0.33
**MSI-H tumor carriers (n=394)**					
	Patients, n	Events, n	Median OS (95%CI), mo	Log-rank p-value	Adj HR (95%CI), p-value
no immunotherapy	261	150	17.0 (13.9-19.5)	$1x10^{-06}$	Ref
immunotherapy at 1st line	102	32	NA (30.2-NA)		0.37 (0.25; 0.56), $2.04x10^{-06}$
immunotherapy at 2nd or later line	31	16	40.1 (31.0-56.2)		0.68 (0.40; 1.16), 0.16
**TTD**					
**All patients (n=8,865)**					
	Patients, n	Events, n	Median TTD (95%CI), mo	Log-rank p-value	Adj HR (95%CI), p-value
no immunotherapy	8,509	6,999	5.3 (5.2-5.3)	0.9	Ref
immunotherapy at 1st line	172	140	4.1 (3.5-5.1)		0.87 (0.72-1.05), 0.15
immunotherapy at 2nd or later line	184	184	5.6 (5.1-6.4)		1.11 (0.94-1.30), 0.22
**MSS tumor carriers (n=8,340)**					
	Patients, n	Events, n	Median TTD (95%CI), mo	Log-rank p-value	Adj HR (95%CI), p-value
no immunotherapy	8,155	6,699	5.7 (5.6-5.9)	0.2	Ref
immunotherapy at 1st line	51	43	3.9 (3.2-6.3)		1.08 (0.79-1.49), 0.62
immunotherapy at 2nd or later line	134	134	6.0 (5.2-7.3)		1.08 (0.89-1.31), 0.45

© 2025 Bari S et al. *JAMA Network Open*.

MSI-H tumor carriers (n=525)

	Patients, n	Events, n	Median TTD (95%CI), mo	Log-rank p-value	Adj HR (95%CI), p-value
no immunotherapy	354	300	4.6 (4.1-5.2)	0.09	Ref
immunotherapy at 1st line	121	97	5.1 (3.6-5.7)		0.84 (0.67-1.08), 0.17
immunotherapy at 2nd or later line	50	50	5.1 (3.0-6.4)		1.13 (0.83-1.54), 0.42

[1] Number of patients retained in the multivariate models
[2] Sample includes patients diagnosed with metastatic colorectal cancer between January 1, 2015 and December 31, 2019
[3] Sample includes patients with unknown MMR status
[4] Index date was the first episode of any systematic treatment regimen administered on or after the date of metastatic disease being diagnosed
[5] Models were adjusted for gender, stage at initial diagnosis, primary cancer site, MMR status, KRAS mutation status, immunotherapy as first line treatment, immunotherapy plus chemotherapy, ECOG performance status, albumin levels, and antibiotic use
[6] Patients were stratified by the line of therapy where they first received immune checkpoint inhibitors during treatment of mCRC

Abbreviations:
- OS = overall survival
- TTD = time to treatment discontinuation
- HR = hazard ratio
- CI = confidence interval
- MMR = mismatch repair
- MSI-H = microsatellite instable-high
- MSS = microsatellite stable

© 2025 Bari S et al. *JAMA Network Open*.

eTable 8. Univariate models for differences in OS and TTD between subgroups of metastatic colorectal cancer patients treated with immunotherapy

	OS					TTD				
	Patients, n [1]	Events, n (% total) [2]	Median OS, mo	95%CI	P-value [3]	Patients, n	Events, n (% total)	Median TTD, mo	95%CI	P-value
Characteristics	566	302 (53.4)	9.4	7.8-10.5		566	386 (68.2)	2.8	2.4-3.4	
Demographic characteristics										
**Sex**					0.002					0.04
Female	282	139 (49.3)	10.5	8.4-18.4		282	192 (68.1)	3.4	2.8-4.6	
Male	284	163 (57.4)	7.3	5.3-10.0		284	194 (68.3)	2.3	2.1-2.9	
**Race**					0.4					0.2
Asian	13	5 (38.5)	6.3	1.5-NA		13	8 (61.5)	2.1	0.7-NA	
Black or African American	53	32 (60.4)	5.1	2.7-12.1		53	39 (73.6)	1.6	1.1-2.8	
White	392	213 (54.3)	9.5	7.7-12.8		392	266 (67.9)	3.2	2.8-3.7	
**Ethnicity**										
Latino/Hispanic										
non-Latino/non-Hispanic										
Clinical characteristics										
**Age at metastatic diagnosis**					0.4					0.7
<50 y	105	49 (46.7)	10	7.3-NA		105	65 (61.9)	2.8	1.9-4.6	
≥50 y	461	253 (54.9)	8.5	7.1-10.5		461	321 (69.6)	2.8	2.4-3.4	
**Practice type**					0.8					0.9
Community	534	284 (53.2)	9.2	7.7-10.5		534	363 (68)	2.8	2.4-3.4	
Academic	32	18 (56.3)	11.5	5.4-NA		32	23 (71.9)	3.4	1.4-14	
**Disease stage at initial diagnosis** [4]					$3x10^{-05}$					$4x10^{-06}$
I-III	286	137 (47.9)	12.8	10.0-22.3		286	181 (63.3)	4.1	3.3-5.5	
IV	251	150 (59.8)	6.3	4.5-8.3		251	185 (73.7)	2.1	1.6-2.7	
**Primary cancer site**					$2x10^{-04}$					0.004
Colon	459	234 (51)	10	8.4-14.1		459	305 (66.4)	3.2	2.7-3.7	
Rectum	101	67 (66.3)	4.8	100-244		101	78 (77.2)	2.1	1.4-3.2	
Tumor characteristics										
**MMR status**					$3x10^{-11}$					$5x10^{-15}$

© 2025 Bari S et al. *JAMA Network Open*.

MSS	235	149 (63.4)	4.4	3.7-6.6		235	186 (79.1)	1.8	1.4-2.3	
MSI-H	234	89 (38)	22.7	15.9-NA		234	127 (54.3)	7.4	4.4-12.5	
***KRAS* mutation status**					0.004					0.009
Wild-type	265	125 (47.2)	10.9	9.0-17.3		265	168 (63.4)	3.4	2.8-4.4	
Positive	217	134 (61.8)	6.4	4.3-9.2		217	164 (75.6)	2.2	1.8-2.8	
***BRAF* mutation status**				-	0.1					0.2
Wild-type	286	145 (50.7)	9.5	6.7-15.0		286	188 (65.7)	2.8	2.3-3.4	
Positive	99	44 (44.4)	17.2	9.5-NA		99	63 (63.6)	4.3	2.7-8	
***NRAS* mutation status**					0.5					0.1
Wild-type	339	164 (48.4)	10.2	9.0-15.9		339	214 (63.1)	3.2	2.8-4.1	
Positive	15	9 (60)	3.4	1.6-NA		15	13 (86.7)	1.6	1.4-NA	
Treatment patterns										
**Immunotherapy as first treatment**					0.004					0.009
No	438	251 (57.3)	8.3	6.4-9.8		438	312 (71.2)	2.8	2.3-3.2	
Yes	128	51 (39.8)	20.1	10.1-NA		128	74 (57.8)	4.1	2.8-7.8	
**Immunotherapy plus chemotherapy** [5]					0.7					0.001
No	469	250 (53.3)	8.8	7.1-10.5		469	308 (65.7)	3.2	2.7-3.7	
Yes	97	52 (53.6)	10	7.7-15.4		97	78 (80.4)	2.1	1.4-3	
**ECOG status** [6]					0.004					0.2
0-1	356	179 (57.4)	10.2	8.5-14.6		356	238 (66.9)	3.2	2.7-3.7	
2-4	94	61 (64.9)	5.5	3.1-8.5		94	67 (71.3)	2.3	1.4-3.7	
**Albumin levels** [6]					$2x10^{-16}$					$2x10^{-06}$
<3 g/dL	83	64 (77.1)	2	1.6-2.6		83	65 (78.3)	0.9	0.7-1.4	
≥3 g/dL	456	227 (49.8)	10.5	9.5-15.2		456	304 (66.7)	3.3	2.8-3.7	
**Antibiotic use** [7]					$4x10^{-04}$					0.02
No	389	215 (55.3)	7.3	5.5-10		389	262 (67.4)	2.5	2.1-3.0	
Yes	177	87 (49.2)	13.4	9.5-24.4		177	124 (70.1)	4.2	3.0-5.0	
**PPI use** [7]					0.5					0.9
No	482	252 (52.3)	9.2	7.9-11.1		482	325 (67.4)	2.8	2.5-3.4	

© 2025 Bari S et al. *JAMA Network Open*.

Yes	82	50 (61)	9.4	4.8-17.6	82	61 (74.4)	2.8	1.8-4.4

[1] Sample includes metastatic colorectal cancer patients who received immune checkpoint inhibitors between January 1, 2015 and December 31, 2019

[2] Index date was the start of ICI-based therapy defined as the first administration or non-cancelled order of any of the following drugs: nivolumab, pembrolizumab, atezolizumab, ipilimumab, tremelimumab, durvalumab, or avelumab

[3] Log-rank p-value

[4] All patients in the cohort were either diagnosed with metastatic colorectal cancer or had progressed to metastatic disease after early-stage diagnosis

[5] Immune checkpoint inhibitors given in combination with chemotherapeutic agents in the same line of therapy

[6] ECOG status and albumin levels were recorded 3 months before or after immunotherapy initiation

[7] Antibiotics or PPIs taken from one month before the start of immunotherapy until the end

Abbreviations:

OS = overall survival
TTD = time to treatment discontinuation
MMR = mismatch repair
MSI-H = microsatellite instable
MSS = microsatellite stable
ECOG = Eastern Cooperative Oncology Group
PPI = proton pump inhibitor

© 2025 Bari S et al. *JAMA Network Open*.

eTable 9. Multivariable Cox proportional hazards models for differences in OS and TTD among metastatic colorectal cancer patients treated with immunotherapy (only including patients diagnosed after May 2017)

	OS											
	All patients (48 event/128 total) [1,2,3]				MSI-H (28 event/91 total)				MSS (20 event/37 total)			
Characteristics	Patients, n	Events, n (% total) [4]	Adj HR (95%CI) [5]	p-value	Patients, n	Events, n (% total)	Adj HR (95%CI)	p-value	Patients, n	Events, n (% total)	Adj HR (95%CI)	p-value
**Sex**				0.66				0.062				0.3
Female	65	21 (43.8)	1 (Ref)		49	10 (35.7)	1 (Ref)		16	11 (55.0)	1 (Ref)	
Male	63	27 (56.2)	1.14 (0.63; 2.09)		42	18 (64.3)	2.36 (0.96; 5.82)		21	9 (45.0)	0.56 (0.19; 1.67)	
**Disease stage at initial diagnosis** [6]				0.11				0.33				0.44
I-III	80	29 (60.4)	1 (Ref)		58	18 (64.3)	1 (Ref)		22	11 (55.0)	1 (Ref)	
IV	48	19 (39.6)	1.65 (0.89; 3.05)		33	10 (35.7)	1.51 (0.66; 3.45)		15	9 (45.0)	1.66 (0.45; 6.06)	
**Primary site**				0.19				0.045				0.86
Colon	115	40 (83.3)	1 (Ref)		88	26 (92.9)	1 (Ref)		27	14 (70.0)	1 (Ref)	
Rectum	13	8 (16.7)	1.93 (0.72; 5.17)		3	2 (7.1)	5.38 (1.04; 27.76)		10	6 (30.0)	0.88 (0.21; 3.71)	
**MMR status**				0.034				–				–
MSS	37	20 (41.7)	1 (Ref)		–	–	1 (Ref)		–	–	1 (Ref)	
MSI-H	91	28 (58.3)	0.43 (0.2; 0.94)		–	–	–		–	–	–	
***KRAS* status**				0.11				0.26				0.51
Wild-type	85	26 (54.2)	1 (Ref)		69	19 (67.9)	1 (Ref)		16	7 (35.0)	1 (Ref)	
Positive	43	22 (45.8)	1.72 (0.88; 3.36)		22	9 (32.1)	1.66 (0.69; 4.03)		21	13 (65.0)	1.57 (0.42; 5.88)	
**Immunotherapy as first treatment**				0.24				0.16				0.46
No	85	37 (77.1)	1 (Ref)		54	19 (67.9)	1 (Ref)		31	18 (90.0)	1 (Ref)	
Yes	43	11 (22.9)	0.63 (0.3; 1.35)		37	9 (32.1)	0.54 (0.23; 1.27)		6	2 (10.0)	0.46 (0.06; 3.71)	
**ECOG status** [7]				0.19				0.015				0.83
0-1	92	30 (62.5)	1 (Ref)		64	16 (57.1)	1 (Ref)		28	14 (70.0)	1 (Ref)	
2-4	36	18 (37.5)	1.64 (0.79; 3.41)		27	12 (42.9)	3.77 (1.29; 11.02)		9	6 (30.0)	0.86 (0.22; 3.39)	
**Albumin level** [7]				0.042				0.64				0.0027
<3g/dL	22	12 (25.0)	1 (Ref)		17	7 (25.0)	1 (Ref)		5	5 (25.0)	1 (Ref)	
≥3g/dL	106	36 (75.0)	0.43 (0.19; 0.97)		74	21 (75.0)	1.33 (0.4; 4.45)		32	15 (75.0)	0.06 (0.01; 0.38)	
**Antibiotic use** [8]				0.0062				0.048				0.0087

© 2025 Bari S et al. *JAMA Network Open*.

No	91	40 (83.3)	1 (Ref)		66	24 (85.7)	1 (Ref)		25	16 (80.0)	1 (Ref)	
Yes	37	8 (16.7)	0.33 (0.15; 0.73)		25	4 (14.3)	0.31 (0.1; 0.99)		12	4 (20.0)	0.14 (0.03; 0.61)	

TTD

	All patients (77 event/146 total)				MSI-H (48 event/106 total)				MSS (29 event/40 total)			
	Patients, n	Events, n (% total)	Adj HR (95%CI) [9]	p-value	Patients, n	Events, n (% total)	Adj HR (95%CI)	p-value	Patients, n	Events, n (% total)	Adj HR (95%CI)	p-value
**Sex**				0.99				0.38				0.091
Female	78	38 (49.4)	1 (Ref)		59	22 (45.8)	1 (Ref)		19	16 (55.2)	1 (Ref)	
Male	68	39 (50.6)	1.00 (0.62; 1.61)		47	26 (54.2)	1.32 (0.71; 2.44)		21	13 (44.8)	0.48 (0.20; 1.12)	
**Disease stage at initial diagnosis**				0.064				0.36				0.16
I-III	89	46 (59.7)	1 (Ref)		67	31 (64.6)	1 (Ref)		22	15 (51.7)	1 (Ref)	
IV	57	31 (40.3)	1.62 (0.97; 2.71)		39	17 (35.4)	1.35 (0.71; 2.57)		18	14 (48.3)	2.11 (0.75; 5.94)	
**Primary site**				0.92				0.39				0.88
Colon	131	67 (87.0)	1 (Ref)		102	46 (95.8)	1 (Ref)		29	21 (72.4)	1 (Ref)	
Rectum	15	10 (13.0)	1.04 (0.45; 2.43)		4	2 (4.2)	1.93 (0.42; 8.81)		11	8 (27.6)	0.91 (0.28; 3.01)	
**MMR status**				0.0097				–				–
MSS	40	29 (37.7)	1 (Ref)		–	–	1 (Ref)		–	–	1 (Ref)	
MSI-H	106	48 (62.3)	0.44 (0.24; 0.82)		–	–	–		–	–	–	
***KRAS* status**				0.058				0.18				0.24
Wild-type	99	46 (59.7)	1 (Ref)		82	35 (72.9)	1 (Ref)		17	11 (37.9)	1 (Ref)	
Positive	47	31 (40.3)	1.67 (0.98; 2.82)		24	13 (27.1)	1.58 (0.81; 3.09)		23	18 (62.1)	1.89 (0.66; 5.40)	
**Immunotherapy as first treatment**				0.65				0.81				0.8
No	98	52 (67.5)	1 (Ref)		64	27 (56.2)	1 (Ref)		34	25 (86.2)	1 (Ref)	
Yes	48	25 (32.5)	1.13 (0.66; 1.95)		42	21 (43.8)	0.92 (0.47; 1.80)		6	4 (13.8)	0.86 (0.26; 2.86)	
**Immunotherapy plus chemotherapy** [10]				0.18				0.036				0.54
No	130	63 (81.8)	1 (Ref)		100	42 (87.5)	1 (Ref)		30	21 (72.4)	1 (Ref)	
Yes	16	14 (18.2)	1.61 (0.81; 3.23)		6	6 (12.5)	3.12 (1.08; 9.04)		10	8 (27.6)	1.36 (0.50; 3.74)	
**Albumin level**				0.11				0.91				0.002
<3g/dL	25	14 (18.2)	1 (Ref)		20	9 (18.8)	1 (Ref)		5	5 (17.2)	1 (Ref)	
≥3g/dL	121	63 (81.8)	0.60 (0.32; 1.11)		86	39 (81.2)	1.05 (0.46; 2.38)		35	24 (82.8)	0.16 (0.05; 0.51)	
**Antibiotic use**				0.06				0.11				0.22

© 2025 Bari S et al. *JAMA Network Open*.

No	105	57 (74.0)	1 (Ref)	78	38 (79.2)	1 (Ref)	27	19 (65.5)	1 (Ref)
Yes	41	20 (26.0)	0.59 (0.34; 1.02)	28	10 (20.8)	0.53 (0.24; 1.16)	13	10 (34.5)	0.52 (0.19; 1.48)

[1] Number of patients retained in the multivariate models
[2] Sample includes patients diagnosed after FDA approval of immune checkpoint inhibitors for treatment of MSI-H metastatic colorectal cancer (May 2017)
[3] Sample includes patients with unknown MMR status
[4] Index date was the start of ICI-based therapy defined as the first administration or non-cancelled order of any of the following drugs: nivolumab, pembrolizumab, atezolizumab, ipilimumab, tremelimumab, durvalumab, or avelumab
[5] For OS, models were adjusted for gender, stage at initial diagnosis, primary cancer site, MMR status, KRAS mutation status, immunotherapy as first-line treatment, ECOG performance status, albumin levels, and antibiotic use
[6] All patients in the cohort were either diagnosed with metastatic colorectal cancer or had progressed to metastatic disease after early-stage diagnosis
[7] ECOG status and albumin levels were recorded 3 months before or after immunotherapy initiation
[8] Antibiotics or PPIs taken anytime from one month before the start of immunotherapy until the end
[9] For TTD, models were adjusted for gender, stage at initial diagnosis, primary cancer site, MMR status, KRAS mutation status, immunotherapy as first-line treatment, immunotherapy plus chemotherapy, albumin levels, and antibiotic use
[10] Immune checkpoint inhibitors given in combination with chemotherapeutic agents in the same line of therapy

Abbreviations:
OS = overall survival
TTD = time to treatment discontinuation
MMR = mismatch repair
MSI-H = microsatellite instable
MSS = microsatellite stable
HR = hazard ratio
CI = confidence interval
ECOG = Eastern Cooperative Oncology Group
PPI = proton pump inhibitor

© 2025 Bari S et al. *JAMA Network Open*.

Supplementary Figures

eFigure. Study Flow Diagram

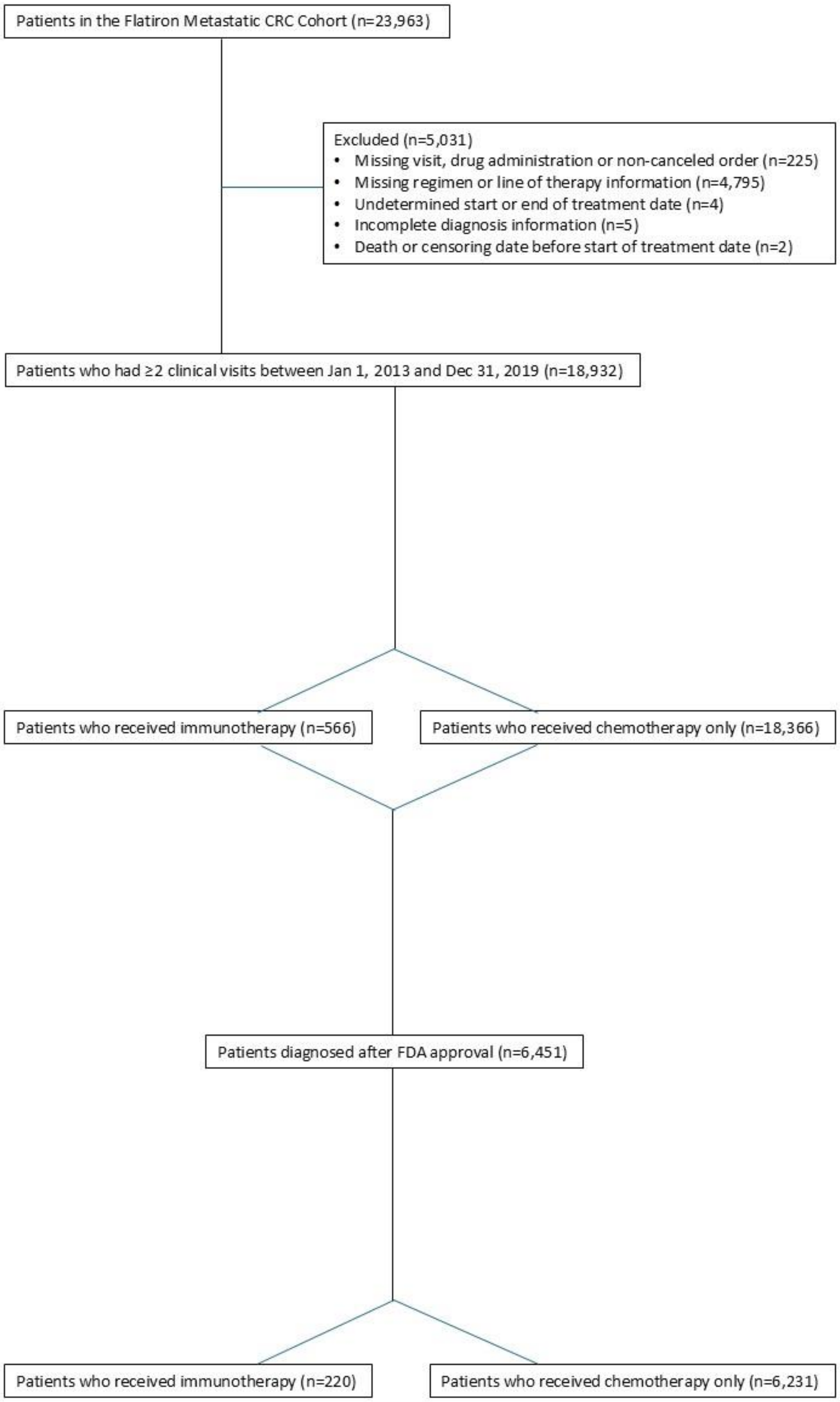

© 2025 Bari S et al. *JAMA Network Open*.

Legend eFigure

Each patient had a diagnosis of metastatic colorectal cancer (ICD-9: 153.x, 154.x; ICD-10: C18x, C19x, C20x, C21x) and at least two documented clinical visits between Jan 2013 and Dec 2019. Sensitivity analyses were carried out on patients diagnosed after FDA approval of immune checkpoint inhibitors for treatment of MSI-H metastatic colorectal cancer (May 2017)**.**

© 2025 Bari S et al. *JAMA Network Open*.
