# Supplementary material for: Practice Patterns and Survival Outcomes of Immunotherapy for Metastatic Colorectal Cancer
Source: JAMA Netw Open. 2025 Mar 20;8(3):e251186. doi: 10.1001/jamanetworkopen.2025.1186 (PMC11926646; doi:10.1001/jamanetworkopen.2025.1186)
Supplement: Supplement 2. — Data Sharing Statement [file jamanetwopen-e251186-s002.pdf]

Data Sharing Statement

Bari. Practice Patterns and Survival Outcomes of Immunotherapy for Metastatic Colorectal Cancer. *JAMA Netw Open*. Published March 20, 2025.
doi:10.1001/jamanetworkopen.2025.1186

Data
Data available: No
